# Synthetic Dual-Input Hybrid Riboswitches—Optimized Genetic Regulators in Yeast

**DOI:** 10.1021/acssynbio.4c00660

**Published:** 2025-02-04

**Authors:** Daniel Kelvin, Janette Arias Rodriguez, Ann-Christin Groher, Kiara Petras, Beatrix Suess

**Affiliations:** † Fachbereich Biologie, TU Darmstadt, Schnittspahnstrasse 10, 64287 Darmstadt, Germany; ‡ Centre for Synthetic Biology, TU Darmstadt, 64287 Darmstadt, Germany

**Keywords:** aptamer, riboswitch, tandem constructs, hybrid riboswitch, dual input, synthetic biology

## Abstract

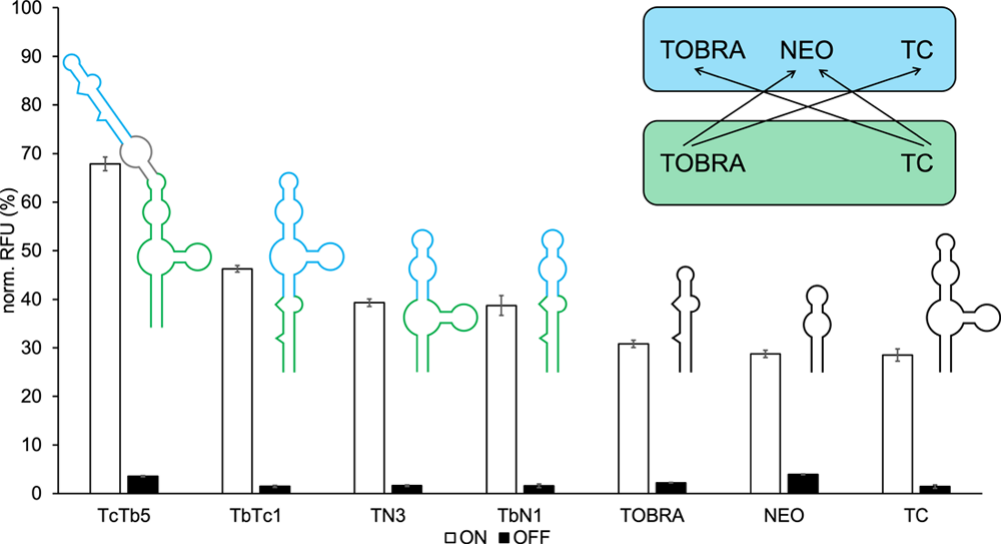

Synthetic riboswitches, genetic regulatory elements composed entirely of RNA, have been engineered to control a variety of mechanisms at the level of both transcription and translation in all domains of life. The efficiency of riboswitch regulation can be increased by inserting two of them into an mRNA sequence in close proximity, resulting in a tandem riboswitch. The tandem state results in improved regulation beyond that of a single riboswitch by allowing both binding pockets to contribute to a higher dynamic range. The focus of this study was to create a novel tandem riboswitch design by integrating the binding pockets of two different riboswitches into one continuous structure, thereby creating a dual-input hybrid riboswitch. These hybrids remain compact in size with a shorter sequence length compared to a tandem riboswitch, while taking advantage of the binding pockets and scaffold sequences provided by both parental riboswitches. Through rational design, hybrid constructs derived from the combination of tetracycline-, tobramycin-, neomycin-, and paromomycin-binding riboswitches were engineered that significantly increase the dynamic range (e.g., from 14- to 36-fold for tobramycin) while increasing their expression levels in the absence of ligand (e.g., 28% to 68% expression for tetracycline). This study expands the toolbox of synthetic riboswitches and establishes general design guidelines applicable to similar riboswitches. Additionally, the dual-input state makes hybrid riboswitches an interesting target for the design of genetic regulators following Boolean logic.

## 1 Introduction

Riboswitches are genetic elements comprised solely of RNA that were originally discovered in the 5′ UTR of bacterial mRNAs.^1^ Riboswitches fold into complex tertiary structures capable of binding a target molecule (ligand) with high specificity and affinity up to the picomolar range.^2^ A riboswitch contains two important domains: the binding domain (aptamer) and an expression platform. The expression platform undergoes structural changes upon binding of the ligand to the aptamer, leading to changes in gene expression. Riboswitch control mechanisms include transcription termination, sequestration of the ribosomal binding site or a splice site, or ribozyme-mediated degradation.^1,3−6^ Up to 55 different classes have been discovered in nature.^7,8^

Control of gene expression by riboswitches requires no additional factors (e.g., repressor proteins). The low metabolic burden on the host cell combined with the compact size of riboswitches (typically 50–100 nucleotides) make them optimal candidates for implementation in complex genetic circuits. Synthetic riboswitches have been developed for use in all domains of life.^9−11^ New mechanisms not yet discovered in nature have been explored through de novo engineering.

In yeast, insertion of aptamers into the 5′ UTR of an mRNA enables control of translation initiation by creating a physical “roadblock” for the scanning ribosome when the ligand is bound.^12,13^ This type of riboswitch requires aptamers with the ability to stabilize their structure upon ligand binding. Selecting for riboswitches capable of performing the roadblock mechanism in yeast has proven effective, as several candidates have been discovered in recent years.^14−18^

Aptamer domains can be selected in vitro against a wide range of potential targets, especially small water-soluble molecules and proteins. The process of selection is known as Systematic Evolution of Ligands by EXponential enrichment (SELEX).^19,20^ During this process the target molecule is immobilized and brought into contact with a large pool of partially randomized RNA oligonucleotides. Wash steps are used to remove nonbinding sequences and potential binders are specifically eluted and enriched. After several iterative rounds, RNA sequences with high affinity for the target molecule can be discovered in the enriched pool. A subsequent screening step in yeast is important to identify aptamers eligible for the riboswitch engineering process.^21^

Riboswitch performance can be tuned by inserting multiple copies of a construct. This design strategy was shown to result in greatly increased regulation efficiency of the roadblock mechanism in yeast.^22,23^ Instead of using identical copies, two different riboswitches can also be inserted in tandem. This allows for improved regulation and a response to different input signals.^16^ Dual-input tandem riboswitches have also been observed in nature. The SAM-AdoCbl tandem riboswitch discovered in *Bacillus clausii* contains two individual riboswitch domains responsive to *S*-adenosylmethionine (SAM) and coenzyme B_12_ (AdoCbl) and is responsible for regulating the expression of *metE*. It encodes for MetE, an enzyme involved in the biosynthesis of methionine.^24^ The dual-input nature of the SAM-AdoCbl tandem riboswitch allows it to act as a Boolean NOR logic gate, thereby optimizing the metabolic pathway of methionine biosynthesis. A synthetically designed tandem riboswitch, resulting from the combination of a neomycin- and a tetracycline-responsive riboswitch, also exhibited a Boolean NOR gate switching pattern in yeast using the roadblock mechanism.^25^

The increase in dynamic range (ratio between unbound and ligand-bound riboswitch-mediated gene expression) and the ability to emulate Boolean logic gates make dual-input riboswitches a favorable design choice for the optimization of riboswitches utilizing the roadblock mechanism in yeast. However, current tandem riboswitch designs suffer from sequence context dependency, requiring time-consuming adjustments to position them in a compatible arrangement. The goal of this study was to improve the dual-input riboswitch concept by combining the binding pockets of two riboswitches into one continuous structure, instead of inserting their full-length sequences separately into the mRNA. This new type of dual-input regulatory genetic element has been termed a hybrid riboswitch. Hybrid riboswitches are designed to surpass the regulatory capabilities of single-input riboswitches, while maintaining their advantages as small, compact and low footprint genetic regulators requiring no additional auxiliary factors. Using binding pockets for two different ligands additionally enables the possibility of using hybrid riboswitches as Boolean logic gates.

Our goal was to demonstrate that a simple plug-and-play approach can be used to design hybrid constructs capable of outperforming established and well-researched small molecule binding riboswitches in both basal expression and dynamic range. We used both rational design and screening strategies to create a toolbox of dual-input hybrid riboswitches that expand the range of highly efficient regulatory elements available for genetic circuit design in yeast.

## 2 Results

### 2.1 Selection of Parental Single-Input Riboswitches

**Figure 1:**
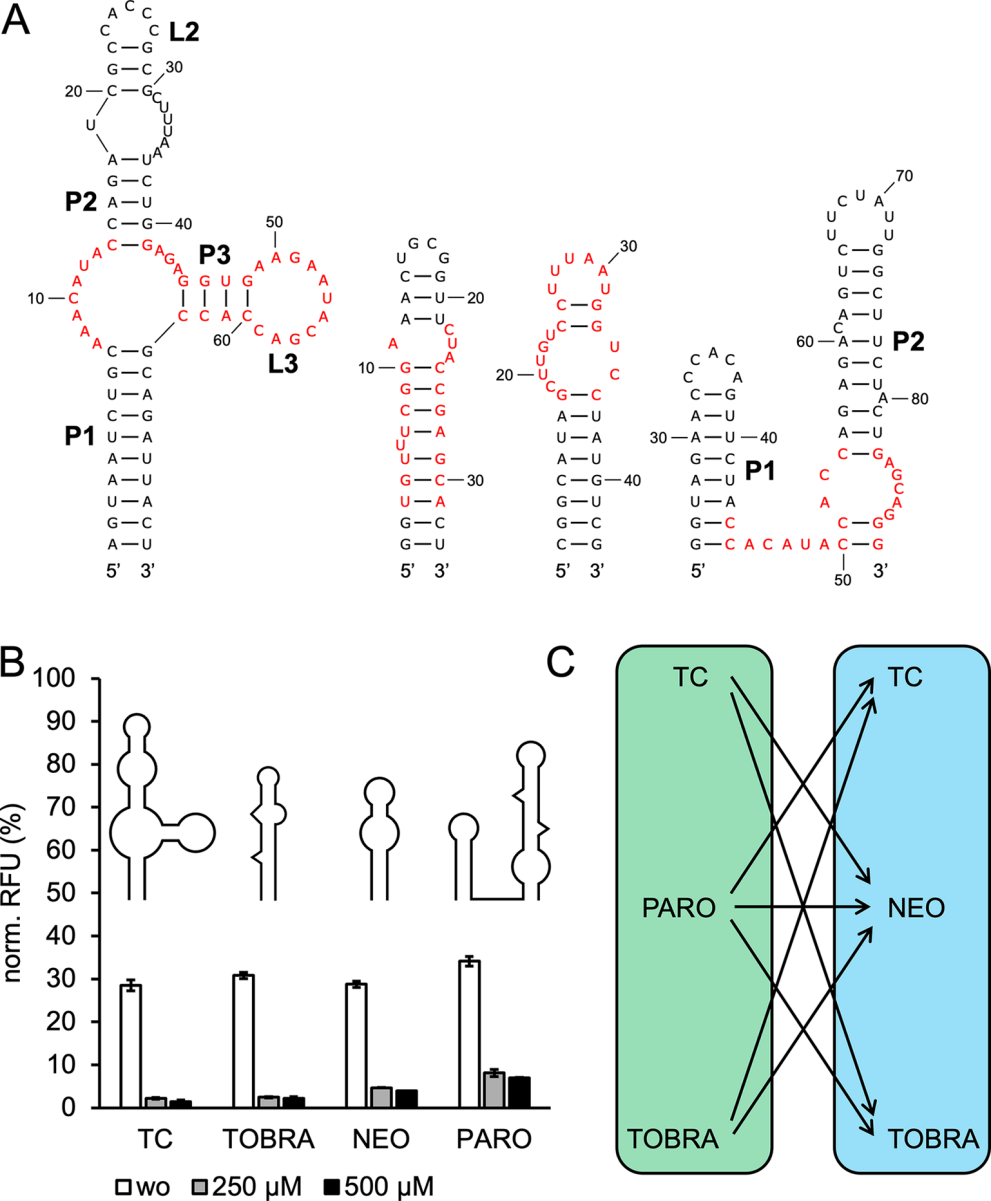
(A) 2D structures of the optimized single-input parental riboswitches chosen for hybrid riboswitch combination (left to right: TC G6, TOBRA N6G5, NEO M4, PARO). Sequence areas containing the binding pockets are marked in red. (B) Measurements of parental riboswitch influence on GFP expression in the absence (wo) or presence of their respective ligands (250 and 500 μM ligand concentration). *S. cerevisiae* RS453 were transformed with the plasmid pCBB06 containing the respective riboswitch in the 5′ UTR of gfp directly upstream of the start codon. GFP fluorescence was normalized to the fluorescence of constitutively expressed mCherry. pCBB06 without a riboswitch served as a blank and its GFP fluorescence was subtracted from all other measurements. The positive control under the same ligand condition as each measurement was set to 100%. Measurements were performed in duplicates and repeated twice. (C) Schematic representation of all parental riboswitch combinations resulting in functional hybrid riboswitches. Riboswitches that could be used as the base of a hybrid are shown on the left side in green (TC, PARO, TOBRA), while those donating the upper half of a hybrid are shown on the right side in blue (TC, NEO, TOBRA).

Four well-characterized synthetic riboswitches have been selected as sequence donors for the design of hybrid riboswitches.^14−17^ The secondary structures of the tetracycline-binding riboswitch (TC), tobramycin-binding riboswitch (TOBRA), neomycin-binding riboswitch (NEO) and paromomycin-binding riboswitch (PARO) are shown in Figure 1A. Each parental riboswitch contains a highly conserved binding pocket (marked in red in Figure 1A) surrounded by scaffolding structures that provide stability to its architecture. The sequences of these scaffolds can be modified or exchanged, leading to changes in overall structure stability, thereby modulating the regulatory activity of the riboswitch. For this study, optimized variants of established riboswitches were chosen or in some cases created, which will be presented in more detail below.

TC has a Y-shaped structure consisting of the basal stem P1 and the stems P2 and P3 connected by the internal bulges B1–2 and B2–3. Stems P2 and P3 are closed off by their respective loops L2 and L3. Nucleotide positions essential for ligand binding reside in B1–2, B2–3 and L3.^26−28^ Several P1 stem variants of TC exist, as changes in its sequence have been shown to impact switching behavior.^23^ The TC P1 stem variants G6, F5,^23^ cb32^13^ and cb32_5 (created for this study), which have proven to be highly efficient, were used in this project (Figure S1A).

TOBRA adopts a hairpin loop structure with multiple internal bulges.^14^ The sequence is conserved to a large extent, with only the terminal loop and adjacent stem sequence being freely interchangeable. The first two base pairs in the closing region of TOBRA can also be modified, but at the expense of the efficiency of the riboswitch. TOBRA N6G5 and its variant N8B5 with a less stable terminal loop were both used for the design of hybrid riboswitches (Figure S1B).

NEO also adopts a hairpin loop structure with a large internal bulge directly below the terminal loop.^15^ The loop and bulge together make up the binding pocket of NEO and are highly conserved. As a consequence, a start codon located in the terminal loop cannot be removed. However, we have shown in earlier studies that this start codon is interestingly not recognized, probably because it is so closely involved in structure formation.^29−31^ The basal stem of NEO can be modified, leading to changes in expression levels both in the absence and presence of a ligand. The two stem variants M4 and M7 were used in this study (Figure S1C).

PARO is the only candidate that contains two separate hairpin loop structures connected by a short single-stranded linker sequence.^16^ The larger hairpin loop P2 contains the binding pocket in its closing region and is divided into several stem segments separated by single nucleotide bulges. P2 can be severely truncated and its sequence modified above the binding pocket, with terminal loop exchanges having a stronger effect on riboswitch performance than stem length (Figure S1D). The first hairpin P1 can be removed, resulting in an overall increase in expression levels independent of ligand presence. When removing P1, its last two bases were retained in the single-stranded linker sequence, as these positions were shown to influence riboswitch performance during the initial characterization of PARO.^16^

Further modification or removal of the single-stranded linker sequence was tested, but resulted in loss of function (Figure S2).

A comparison of the expression levels controlled by the best performing parental riboswitch variants in the absence and presence of their respective ligands is shown in Figure 1B. The corresponding data can be viewed in Table 1. All riboswitches analyzed in this study were inserted at the same position in a *gfp* reporter gene directly upstream of the start codon located on the plasmid pCBB06.^16^ GFP values were determined and normalized to constitutive mCherry expression. The *mCherry* gene is located on the same plasmid. Effects of single and double ligand conditions on GFP and mCherry fluorescence values were accounted for by using ligand-specific controls for all calculations to avoid an artificial increase of the dynamic range (Figure S3). All chosen parental riboswitches bind their respective ligand with high specificity, showing no significant cross reactivity (Figure S4). Ligand influence on yeast culture growth was also analyzed (Table S1). No significant effect on growth was observed under the tested conditions. An overview of all possible combinations of the parental riboswitches tested in this study is shown in Figure 1C.

**Table 1:** Parental Riboswitches

	relative fluorescence^a^			dynamic range	
Construct	wo	250 μM	500 μM	250 μM	500 μM
TC G6	28.5	2.2	1.4	12.9×	20.4×
TC F5	21.9	1.5	1.1	14.6×	19.9×
TC cb32	31.1	5.5	3.9	5.6×	8.0×
TC cb32_5	43.7	12.7	10.3	3.4×	4.2×
TOBRA N6G5	30.8	2.4	2.2	12.8×	14.0×
TOBRA N8B5	50.6	5.4	4.8	9.4×	10.5×
NEO M4	28.8	4.7	3.9	6.1×	7.4×
NEO M7	23.3	5.4	4.8	4.3×	4.8×
PARO	34.1	8.1	7.0	4.2×	4.9×
PARO S1	71.4	37.3	33.0	1.9×	2.2×
PARO S2	65.6	31.9	28.2	2.1×	2.3×
PARO S3	58.2	29.1	25.2	2.0×	2.3×

a Relative GFP fluorescence of parental riboswitch variants in the absence (wo) and presence of 250 μM or 500 μM of their respective ligand. A background was subtracted (pCBB06) and GFP expression was normalized to mCherry expression. The positive control under the same ligand condition as each measurement was set to 100%.

A side effect of riboswitch insertion is reduced expression of the controlled gene, even in the absence of a ligand (basal expression). It is important to note that basal expression is always impaired by riboswitch insertion into the 5′ UTR compared to expression without an inserted riboswitch. This effect results from partial structure formation (prestructured state) of the riboswitch.^21^ After addition of the ligand, the final structure is adopted. When designing hybrid riboswitches, improvements in dynamic range can be achieved either by increasing basal expression by destabilizing the prestructured state or by decreasing expression in the ligand-bound state. Tight regulation after ligand binding is expected to be achieved through additional structure stabilization provided by the second binding pocket. When designing hybrids, connecting sequences were created by destabilizing the scaffolds of one or both parental riboswitches through truncations or by adding sequence elements (bulges, CAA repeats) that would break up the structure into smaller segments.

### 2.2 Rational Design of PARO Hybrid Riboswitches

The first rational design approach was to combine TC and PARO by replacing the PARO P2 terminal loop with the TC main bulge. Therefore, the entire TC P1 stem was removed and replaced with the PARO P2 stem. This was possible due to the TC P1 stem sequence being completely interchangeable.^26^ The PARO P2 stem thus becomes the connecting sequence between the two binding pockets. Making the top CG base pair of PARO P2 the closing base pair of the TC binding pocket was deemed feasible, due to previous works showing a preference for stronger base pairing in this region of TC.^23^ Additionally, preliminary experiments showed that basal expression increases when utilizing a flexible PARO P2 terminal loop (Figure S1D). The bulge of the TC binding pocket was therefore proposed to be a sufficient replacement for the terminal loop of PARO P2. The P1 stem of PARO was removed to prevent the hybrid from becoming too stable in its prestructured state due to the additional TC stem-loop structures.

**Figure 2:**
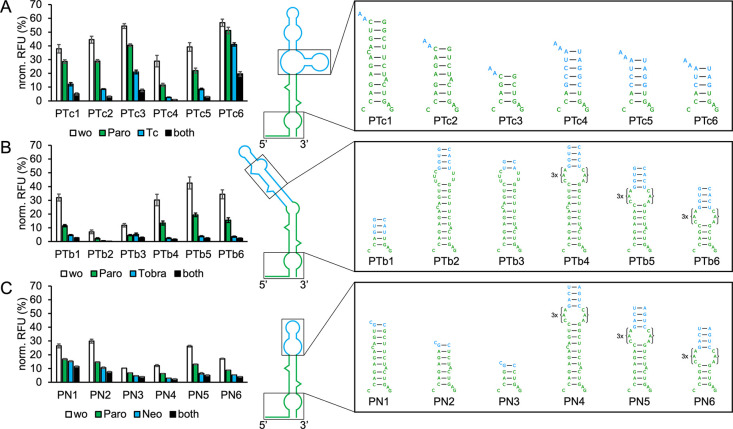
GFP measurements of PARO hybrid riboswitches. (A) PARO-TC, (B) PARO-TOBRA and (C) PARO-NEO hybrid riboswitches were measured in the absence (wo) and presence of 250 μM paromomycin (Paro) and 250 μM (A) tetracycline, (B) tobramycin, or (C) neomycin (Tc; Tobra; Neo) individually and simultaneously (both). A 2D schematic of the hybrids shows areas and connecting stem sequences originating from PARO (green) and (A) TC, (B) TOBRA, or (C) NEO (blue), with the binding pockets being marked by black squares. For all measurements *S. cerevisiae* RS453 were transformed with the plasmid pCBB06 containing the respective hybrid riboswitch in the 5′ UTR of gfp directly upstream of the start codon. GFP fluorescence was normalized to the fluorescence of constitutively expressed mCherry. pCBB06 without a riboswitch served as a blank and its GFP fluorescence was subtracted from all other measurements. The positive control under the same ligand condition as each measurement was set to 100%. Measurements were performed in duplicates and repeated twice.

The predicted secondary structure of the resulting hybrid riboswitch PTc1 is shown in Figure 2A. Its sequence, as well as those of all other hybrid constructs, was inserted directly upstream of the start codon of a *gfp* reporter gene into the vector pCBB06. Yeast cells were transformed with the resulting plasmids and hybrid riboswitch influence on gene expression was measured under all four ligand conditions (no ligand; 250 μM paromomycin; 250 μM tetracycline; 250 μM of both) (Figure 2; Table 2).

**Table 2:** PARO Hybrid Riboswitches

	relative fluorescence^a^		dynamic range at 250 μM		
construct	wo	both	Paro	second ligand	both
PTc1	37.9	5.1	1.3×	3.1×	7.4×
PTc2	44.5	3.1	1.5×	5.2×	14.3×
PTc3	54.4	7.7	1.3×	2.6×	7.1×
PTc4	28.9	1.0	2.5×	11.1×	28.9×
PTc5	39.2	2.9	1.8×	4.6×	13.5×
PTc6	56.8	19.6	1.1×	1.4×	2.9×
PTb1	32.0	2.7	2.8×	6.8×	11.8×
PTb2	7.1	0.3	3.1×	14.2×	23.7×
PTb3	11.9	2.9	2.5×	2.3×	4.1×
PTb4	30.2	1.7	2.2×	11.6×	17.8×
PTb5	42.6	2.6	2.2×	11.0×	16.4×
PTb6	34.4	2.4	2.2×	9.6×	14.3×
PN1	26.4	11.5	1.6×	1.7×	2.3×
PN2	29.9	7.5	2.0×	2.8×	4.0×
PN3	10.3	3.9	1.5×	2.2×	2.6×
PN4	12.1	2.3	1.9×	4.0×	5.3×
PN5	26.2	5.0	2.0×	4.1×	5.2×
PN6	17.1	4.0	2.0×	3.2×	4.3×

a Relative GFP fluorescence of PARO hybrid riboswitches in the absence (wo) and presence of 250 μM of both of their respective ligands. A background was subtracted (pCBB06) and GFP expression was normalized to mCherry expression. The positive control under the same ligand condition as each measurement was set to 100%.

PTc1 showed an increase in basal expression when compared with the parental riboswitches PARO and TC. The dynamic range of PTc1 was however decreased in comparison to the parental riboswitch TC, even in the presence of both ligands (dual-input). Truncations of the connecting PARO P2 stem further increased basal expression levels of the respective constructs PTc2 and PTc3, with PTc2 also exhibiting an increased dynamic range (Table 2). An alternative design used the TC P1 stem variant originating from cb32^13^ as part of the connecting stem. The P1 variant cb32_5 was chosen instead of the G6 variant due to its smaller size (P1 cb32_5:5 bp vs P1 G6:10 bp) to avoid a high degree of prestructuring of the hybrid. By using the full-length TC P1 cb32_5 stem and two truncated versions together with the shortest version of PARO, the three constructs PTc4–6 were created (Figure 2A). PTc4 achieves a 28.9-fold dual-input dynamic range. The hybrid thereby surpasses the dynamic ranges of both parental riboswitches and is even more efficient than the optimized TC G6 variant (20-fold at 500 μM tetracycline), while retaining comparable expression levels. All functional PARO-TC hybrids presented switching behavior in the presence of both ligands individually and simultaneously, with dynamic ranges increasing cumulatively in the presence of both ligands. However, the addition of only paromomycin generally yielded low dynamic ranges.

Combinations of PARO and TOBRA were tested next. The hairpin structure of the TOBRA riboswitch and its higher degree of sequence conservation in comparison to TC made it a more challenging candidate for rational design. Placing TOBRA directly on top of PARO without truncating both constructs as much as possible was not considered feasible due to expected issues with high levels of construct prestructuring. To avoid the occurrence of premature start codons in the connecting sequence of the hybrid, a less stable GU wobble base pair was introduced in between both riboswitches. The resulting construct PTb1 performed similar to TOBRA in both basal expression and dual-input dynamic range, with single-input dynamic ranges underperforming in comparison to the parental riboswitches (structure shown in Figure 2B; data in Table 2).

An alternative design using the PARO L2 terminal loop as a flexible linker by inserting TOBRA directly into its sequence was also tested. The resulting construct PTb2 was still too stable, leading to near complete abolishment of gene expression even in the absence of a ligand. While a truncation of the first two base pairs of TOBRA (PTb3) did result in increased basal expression, a decrease in dynamic range for the TOBRA binding pocket was observed. In an attempt to decrease stability of the hybrid without truncating TOBRA, the terminal loop of the PARO P2 stem was exchanged for a larger artificial linker consisting of three CAA spacers on each site of the connecting region (PTb4). This version of the hybrid yielded basal expression levels comparable to the parental TOBRA riboswitch while achieving an increased 17.8-fold dual-input dynamic range. Truncations of the PARO P2 stem were again tested. Removal of one or both bulge-separated segments of PARO P2 in the respective constructs PTb5 and PTb6 resulted in an additional increase in basal expression levels. PTb5 in particular achieved basal expression levels of over 42% and a 16.4-fold dual-input dynamic range (Figure 2B; Table 2), making it more efficient than its parental riboswitches PARO and TOBRA (Table 1).

The NEO riboswitch is the most compact of all utilized parental riboswitches, with its binding pocket being the highly conserved terminal loop and bulge of the structure. This makes the addition of a second binding pocket on top of NEO not feasible. Similar to PTc1, the first design attempt was to replace the terminal L2 loop of the PARO P2 main stem with the NEO binding pocket. The NEO basal stem was removed to avoid overstabilization, with only the conserved GC closing base pair of the bulge being retained. Variants containing truncations of the PARO P2 stem comparable to PTc2 and PTc3 were also created. The resulting hybrids PN1–3 showed only low dynamic ranges, even in the presence of both ligands (Figure 2C). The best performing construct PN2 had the same PARO P2 stem truncation of the upper bulge-separated segment as PTc2, the most efficiently regulating construct out of PTc1–3. PTb5, the overall best performing PARO-TOBRA hybrid riboswitch, also utilized this PARO P2 stem variant. An indirect connection through a bulge-linker in between PARO and NEO equivalent to the design used for PTb4–6 was also tested, again utilizing three CAA spacers on each site as linkers. The NEO variant M7 was chosen for this design, as M4 contains a premature start codon within its basal stem. Out of the three resulting constructs PN4–6 the highest dual-input dynamic range and basal expression level was observed for PN5, which utilized the same PARO P2 stem truncation as PTc2 and PTb5 (Table 2). None of the designed PARO-NEO riboswitches were capable of outperforming their parental riboswitches, with PN5 only showing a slight improvement in basal expression and dynamic range over the less efficient NEO variant M7. Direct combinations utilizing a fully truncated version of PARO were also tested, but did not present any improvements in comparison to the previously tested hybrid constructs (Figure S5). While the results show a general transferability of rationally designed sequence elements in between the different PARO hybrid combinations, the NEO binding pocket appears to be more sequence-context dependent than TC and TOBRA in its basal/connecting stem sequence. As combinations with a stronger partner than PARO were expected to result in hybrids with higher dynamic ranges, further optimization of PARO-NEO hybrids was not undertaken. Designs attempting to place PARO on top of TC or TOBRA were tested, but failed to produce functional constructs (data not shown).

### 2.3 Rational Design of TC Hybrid Riboswitches

TC was utilized as a base for the rational design of TC-NEO and TC-TOBRA hybrid riboswitches. Knowledge gained from the PARO hybrid riboswitch designs was used to optimize the design strategy. For TC-NEO combinations, the P2 stem of TC was completely exchanged with the NEO riboswitch. Turning the basal stem sequence of NEO into the connecting stem of the hybrid was done in an attempt to alleviate the issues caused by the sequence context dependency of NEO. It was also tested if the stronger NEO M4 variant could be used for the combination of a hybrid riboswitch by inverting a base pair in its basal stem to avoid the occurrence of an additional start codon. The first two base pairs of M4 were truncated to achieve a total stem length of five base pairs (same size as full-length M7), as the full-length NEO M4 basal stem was expected to be too stable to act as the connecting stem. The TC G6 P1 stem variant was chosen due to its strong performance with the parental TC riboswitch.

**Figure 3:**
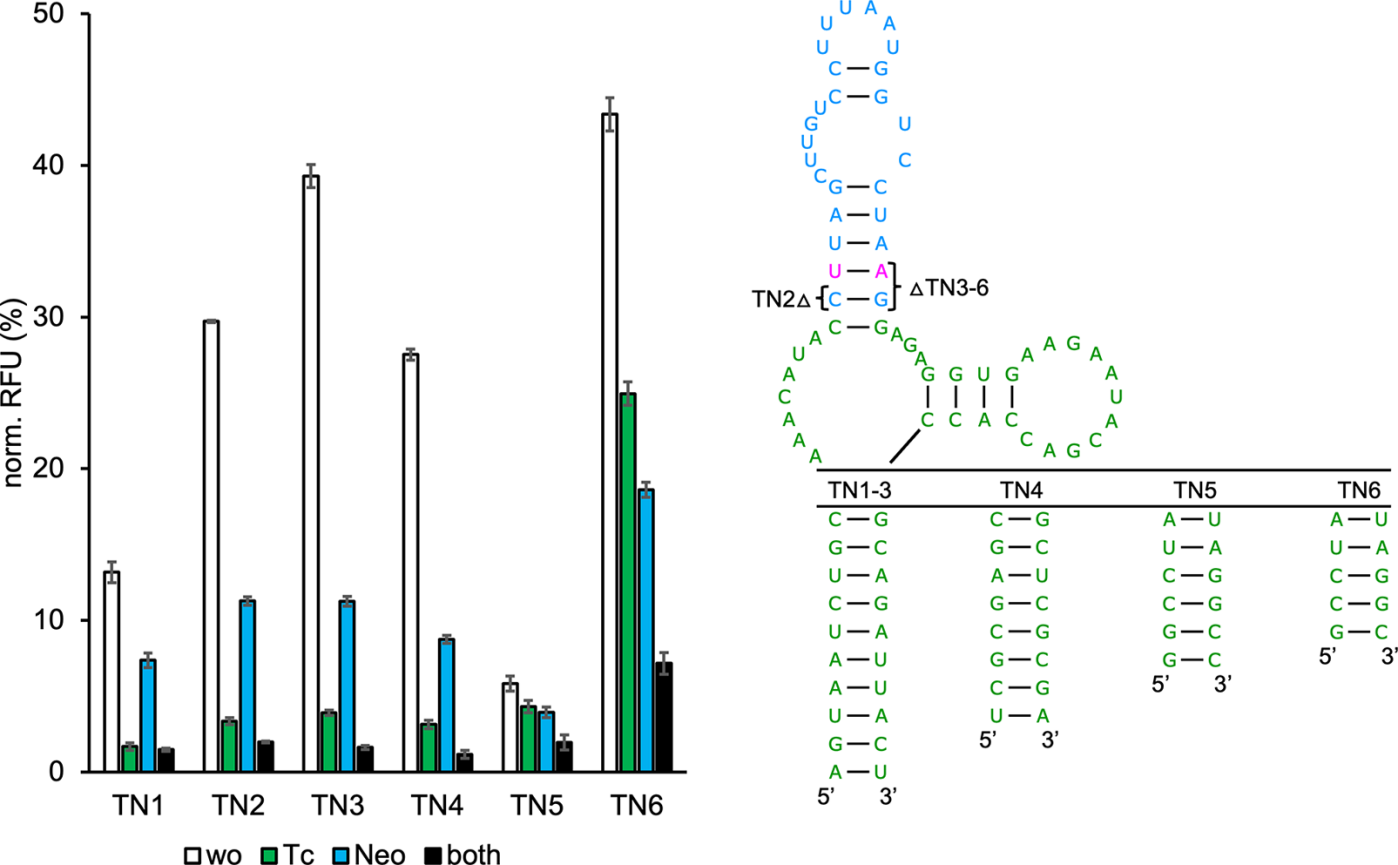
GFP measurements of TC-NEO hybrid riboswitches. Constructs were measured in the absence (wo) and presence of 250 μM tetracycline (Tc) and 250 μM neomycin (Neo) individually and simultaneously (both). The 2D structure of TN1 is shown, with sequences originating from TC being marked in green and sequences originating from NEO M4 being marked in blue. The inverted UA base pair of NEO M4 is marked in red. Truncations of the connecting stem corresponding to other TC-NEO constructs, as well as the TC P1 stem variants used by each construct (TN1–3: TC G6; TN4: TC F5; TN5: TC cb32; TN6: Tc cb32_5) are shown. For all measurements *S. cerevisiae* RS453 were transformed with the plasmid pCBB06 containing the respective hybrid riboswitch in the 5′ UTR of gfp directly upstream of the start codon. GFP fluorescence was normalized to the fluorescence of constitutively expressed mCherry. pCBB06 without a riboswitch served as a blank and its GFP fluorescence was subtracted from all other measurements. The positive control under the same ligand condition as each measurement was set to 100%. Measurements were performed in duplicates and repeated twice.

The resulting construct TN1 (structure shown in Figure 3) presented an overall decrease in basal expression levels when compared to its parental riboswitches. While the dynamic range of NEO in the respective single-input state (1.8-fold; neomycin) remains comparable to results measured for PARO-NEO constructs (Table 2), a 7.8-fold dynamic range in the presence of only tetracycline is reached (Table 3). These results, while originating from an unoptimized candidate, already indicate an increased performance of TC as a base in comparison to PARO. To further increase basal expression and dynamic range, variants with increasingly truncated connecting stems were created (Figure 3). The truncation of one or two additional base pairs from the basal end of the M4 stem led to a strong increase in dynamic range for the constructs TN2 and TN3. While the basal expression levels of TN2 are comparable to TC (29.7% TN2 vs 28.5% TC G6), TN3 reaches close to 40% basal expression and a 24.3-fold dual-input state dynamic range. The single-input dynamic range in the presence of tetracycline also increased to over 10-fold for TN3, reaching values closer to the original TC G6 variant (12.9-fold dynamic range at 250 μM tetracycline). The increased efficiency of TC in comparison to PARO as the base of a hybrid construct was attributed to the P2 stem of TC not affecting the dynamic range of the riboswitch as much as the terminal loop composition of PARO affects its riboswitch. Additionally, TC is the more efficient riboswitch in comparison to PARO. Variations of the TC P1 stem were tested with the optimized communication stem of TN3 to elucidate the effects of TC basal stem composition on hybrid riboswitch behavior. The resulting constructs TN4–6 used the respective stem variations TC F5, TC cb32 and TC cb32_5. The constructs behaved similarly to their single-input TC riboswitch counterparts (Table 1), with TN4 (TC F5) exhibiting a similar dual-input dynamic range to TN3 (TC G6), but at lower basal expression levels, and TN6 (TC cb32_5) exhibiting the highest basal expression and reduced dynamic range. Only the TC P1 cb32 stem behaved different and did not result in a functional hybrid for TN5 (Table 3). TC G6 remains as the best performing P1 stem variant in both the single-input TC riboswitch and the TC-NEO hybrid. The results of the TC-NEO M4 hybrid riboswitches are shown in Figure 3. All presented connecting stem truncations and TC P1 stem variations were also tested with NEO M7. The resulting constructs generally exhibited decreased expression levels, with TC P1 stem variations having similar effects when compared to their NEO M4 counterparts (Figure S6).

**Table 3:** TC Hybrid Riboswitches

	relative fluorescence^a^		dynamic range at 250 μM		
construct	wo	both	Tc	second ligand	both
TN1	13.2	1.5	7.8×	1.8×	8.8×
TN2	29.7	2.0	8.7×	2.6×	14.9×
TN3	39.3	1.6	10.1×	3.5×	24.6×
TN4	27.5	1.2	8.9×	3.1×	22.9×
TN5	5.8	1.9	1.3×	1.5×	2.9×
TN6	43.4	7.2	1.7×	2.3×	6.0×
TcTb1	12.0	0.8	5.7×	8.6×	15.0×
TcTb2	53.8	2.5	6.5×	5.1×	21.5×
TcTb3	71.5	5.1	6.6×	3.0×	14.0×
TcTb4	85.2	7.3	5.8×	3.0×	11.7×
TcTb5	67.8	3.6	8.6×	3.7×	18.8×
TcTb6	46.9	1.3	13.0×	5.2×	36.1×

a Relative GFP fluorescence of TC hybrid riboswitches in the absence (wo) and presence of 250 μM of both of their respective ligands. A background was subtracted (pCBB06) and GFP expression was normalized to mCherry expression. The positive control under the same ligand condition as each measurement was set to 100%.

**Figure 4:**
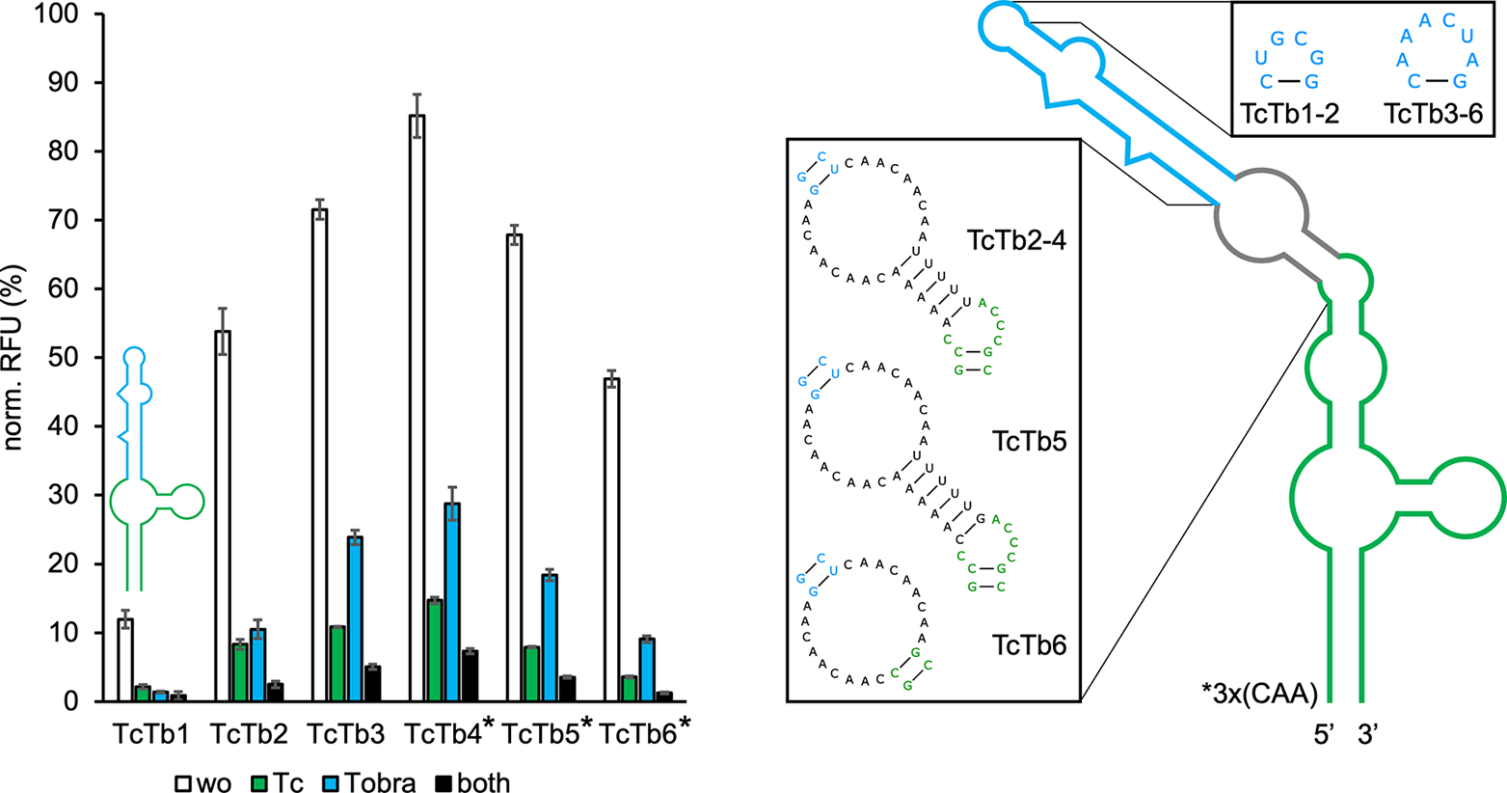
GFP measurements of TC-TOBRA hybrid riboswitches. Constructs were measured in the absence (wo) and presence of 250 μM tetracycline (Tc) and 250 μM tobramycin (Tobra) individually and simultaneously (both). The 2D schematics of TcTb1 (in graph) and TcTb2 are shown, with sequences originating from TC G6 being marked in green and sequences originating from TOBRA being marked in blue. The artificial linker of TbTc2–6 is marked in gray. Truncations of the connecting sequence corresponding to other TC-TOBRA constructs, as well as the TOBRA terminal loop variants used by each construct (TbTc1–2: TOBRA N6G5; TbTc3–6: TOBRA N8B5) are shown. Constructs TcTb4–6 (*) additionally contain 3× CAA spacers upstream of their sequence in the 5′ UTR of gfp. For all measurements *S. cerevisiae* RS453 were transformed with the plasmid pCBB06 containing the respective hybrid riboswitch in the 5′ UTR of gfp directly upstream of the start codon. GFP fluorescence was normalized to the fluorescence of constitutively expressed mCherry. pCBB06 without a riboswitch served as a blank and its GFP fluorescence was subtracted from all other measurements. The positive control under the same ligand condition as each measurement was set to 100%. Measurements were performed in duplicates and repeated twice.

The design approach of directly replacing the TC P2 stem used for TC-NEO hybrid riboswitches was also tested for the combination of TC and TOBRA. The sequence of TOBRA was left unmodified, as previous results had shown that removing the first two base pairs of TOBRA, while improving basal expression, leads to decreased dynamic range. The resulting construct TcTb1, which again utilized the TC G6 P1 basal stem, exhibited only low basal expression levels (12%). The structure of TcTb1 is shown schematically in Figure 4. These results were expected due to both parental riboswitches being highly structured and, when inserted together into an mRNA, having a strong effect on ribosome scanning even in their prestructured states. Using alternative TC P1 stem variants did not alleviate this issue (Figure S7). An alternative design approach utilizing the 3 × CAA-bulge linker from the PARO-TOBRA hybrid riboswitch design was used in an attempt to increase basal expression levels. Instead of replacing the L2 loop of TC with the bulge linker, an additional stem consisting of five A-U base pairs placed on top of L2 was used to connect TC to the bulge. The longer communication sequence (TC P2—L2—5× A-U—3 × CAA—TOBRA) between TC and TOBRA was designed to reduce structuring in the unbound state of the hybrid by creating more spatial separation between the upstream and downstream half of the TC binding pocket. The binding affinities of TC and TOBRA as the two best performing parental riboswitches were expected to be strong enough to enable structure formation of the hybrid in the presence of both ligands. The extended bulge linker was also intended to separate TOBRA from the surrounding sequence context, as shown for PTb4–6. The construct TcTb2 utilized this design together with the TC G6 P1 basal stem to achieve a basal expression of 53.8% and a 21.5-fold dual-input dynamic range (Table 3). The high basal expression and dynamic range were seen as an indication that performance could be increased even further. The terminal loop N6G5 of TOBRA was exchanged for the less stable N8B5 version, resulting in the construct TcTb3. The basal expression of TcTb3 increased to 71.5% with a 14.0-fold dual-input dynamic range. An alternative construct containing the TOBRA N8B5 loop additionally received three more CAA-spacers upstream of the TC P1 basal stem to isolate the entire structure from the sequence context of the 5′ UTR. This construct (TcTb4) reached basal expression levels above 85%, but only a 11.7-fold dual-input dynamic range. In an attempt to balance basal expression and dynamic range, construct TcTb4 with the highest basal expression was stabilized by modifying the connecting 5× AU stem. Addition of another AU base pair resulted in reduced basal expression without a significant increase in dual-input dynamic range (data not shown). Insertion of a G-C base pair at the basal end of the connecting stem enabled the construct TcTb5 to achieve 67.8% basal expression and a 18.8-fold dual-input dynamic range. Another approach to increase the dual-input dynamic range of TcTb4 was to remove the entire connecting stem, which was previously proposed to destabilize the hybrid in its unbound state. This truncation was also done as a control experiment to verify the positive effect of the connecting stem on basal expression. In construct TcTb6 TC and TOBRA N8B5 were connected directly through the 3× CAA bulge linker by removing the 5× AU stem and terminal TC L2 loop. Removal of the 5× AU connecting stem resulted in a strong decrease in basal expression to 46.9% (TcTb4: 85.2%) while the dual-input dynamic range of TcTb6 increased to 36.1-fold (see Table 3 for comparison). The changes in basal expression and dual-input dynamic range between TcTb4 and TcTb6 support the theory behind the rational design approach that the 5× AU connecting stem assists in destabilizing the hybrid structure. The results and secondary structures of all TC-TOBRA hybrid riboswitches can be viewed in Figure 4.

TcTb5 achieves the best balance between basal expression and dual-input dynamic range of all reported hybrids, whereas TcTb6 reaches the highest recorded dual-input dynamic range of the project while maintaining moderate basal expression levels. Both constructs are the overall best performing rationally designed hybrid riboswitches.

An inversion of the design utilizing TOBRA as the base by exchanging its terminal loop with the binding pocket of TC was seen as an alternative design approach toward a more compact version of the hybrid. The short modifiable stem segment of TOBRA between the terminal loop and upper internal bulge would act as the connecting stem, providing stability solely through sequence composition. The above-described hybrid riboswitches utilizing direct connecting stems (PARO-TC; TC-NEO) were successfully designed by truncating the scaffold stems of the respective parental riboswitches. As an alternative design approach for discovering optimal connecting sequences, the connecting stem was randomized and a screening was used to find the best performing candidate. This strategy was chosen for both TOBRA-TC and TOBRA-NEO hybrid riboswitches.

### 2.4 In Vivo Screening for Improved TOBRA Hybrid Riboswitches

Two pools were designed for both TOBRA-TC and TOBRA-NEO containing either three or four base pair long connecting stems in between the upper bulge of TOBRA and the binding pocket of TC and NEO, respectively. The TOBRA-TC pools N6 and N8 contained six and eight randomized positions, respectively. The TOBRA-NEO pools N4 and N6 contained a conserved G-C base pair in the connecting stem below the binding pocket of NEO, resulting in four (N4) or six (N6) randomized positions. Yeast cells were transformed using a high-efficiency electroporation protocol to generate yeast libraries capable of covering all potential combinations [transformation efficiencies: 2.5 × 10^6^ cfu/μg (TbTc_N6); 3 × 10^6^ cfu/μg (TbTc_N8); 1.1 × 10^6^ cfu/μg (TbN_N4); 1.8 × 10^6^ cfu/μg (TbN_N6)]. Fluorescence-based cell sorting was used to enrich functional candidates with a high dynamic range in each pool. GFP fluorescence was normalized to mCherry fluorescence for the entire screening process. The pools were initially sorted for low fluorescence in the presence of both ligands. A second sorting step was used to select for candidates with high fluorescence in the absence of both ligands from the enriched subpopulation of the first round. The entire process was repeated to further enrich desired candidates and remove false-positives. Potential candidates were separated into a 96-well plate (one plate per pool). Fluorescence measurements were performed for all individual candidates in the absence and presence of both ligands and candidates with a high dynamic range were selected. The plasmid DNA of 40 selected candidates from all four pools was purified from the yeast cells and sequenced. Yeast cells were transformed with these plasmids and gene expression was measured again. The entire screening process is schematically presented in Figure S8.

**Figure 5:**
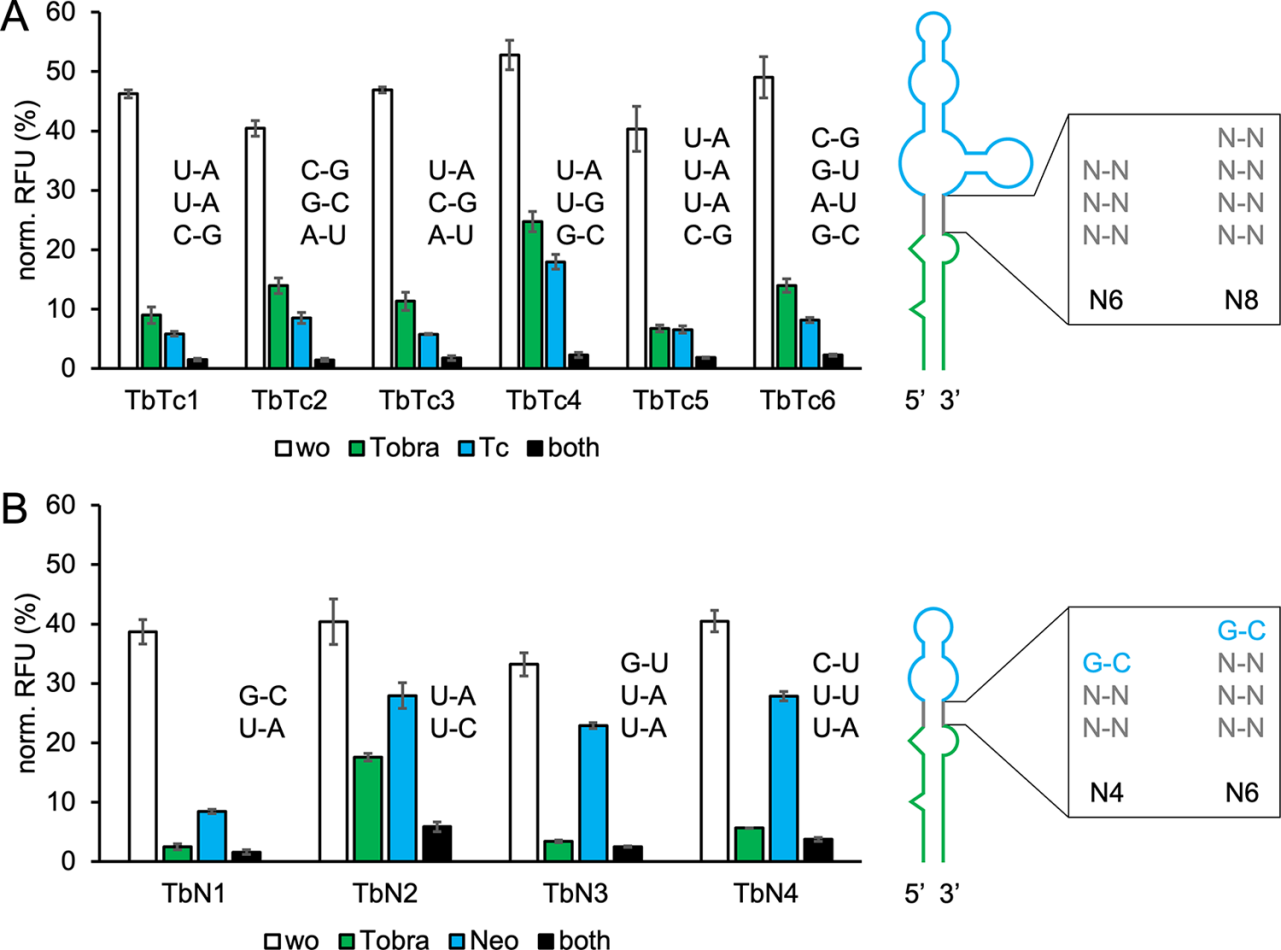
GFP measurements of TOBRA hybrid riboswitches originating from yeast library screenings. Yeast libraries were created by transforming yeast RS453 with linearized pCBB06 and (A) TOBRA-TC N6 or N8, or (B) TOBRA-NEO N4 or N6 pool sequences containing areas homologous to the vector on both ends. TOBRA-NEO randomized connecting sequences had less variability due to the top base pair of the stem being a conserved position of NEO. Electroporation was used to generate yeast libraries with high diversity (transformation efficiency >10^6^ cfu/μg). Yeast libraries were sorted for high GFP fluorescence in the absence of both ligands and afterward for low GFP fluorescence in the presence of both ligands, respectively. Both sorting steps were repeated once to enhance enrichment of functional candidates. During the entire sorting process GFP fluorescence was normalized to mCherry fluorescence. A 96-well plate screening was used to measure individual candidates from the enriched libraries in the absence and presence of both ligands. Candidates with a high dynamic range were chosen and sequenced. Chosen candidates were remeasured in 24-well plates in the absence (wo) and presence of 250 μM tobramycin (Tobra) and 250 μM (A) tetracycline (Tc), or (B) neomycin (Neo) individually and simultaneously (both). For all 24-well plate measurements *S. cerevisiae* RS453 were transformed with the plasmid pCBB06 containing the respective hybrid riboswitch in the 5′ UTR of gfp directly upstream of the start codon. GFP fluorescence was normalized to the fluorescence of constitutively expressed mCherry. pCBB06 without a riboswitch served as a blank and its GFP fluorescence was subtracted from all other measurements. The positive control under the same ligand condition as each measurement was set to 100%. Measurements were performed in duplicates and repeated twice.

Several TOBRA-TC candidates achieved over 20-fold dual-input dynamic ranges (Table 4). TbTc1 (N6D7) reached a 30.9-fold dual-input dynamic range and 46.3% basal expression, making it the second best hybrid riboswitch after TcTb6. Out of all TOBRA-TC candidates TbTc4 (N6G11) contained the only randomized connecting stem sequence discovered multiple times (2×). TOBRA-TC hybrids originating from pool N8 on average performed less efficient with TbTc5 (N8B9) and TbTc6 (N8C3) being the only candidates to reach a higher than 20-fold dual-input dynamic range (Figure 5A).

**Table 4:** TOBRA Hybrid Riboswitches

	relative fluorescence^a^		dynamic range at 250 μM		
construct	wo	both	Tobra	second ligand	both
TbTc1	46.3	1.5	5.1×	7.8×	30.9×
TbTc2	40.5	1.5	2.9×	4.8×	27.0×
TbTc3	46.9	1.8	4.2×	8.1×	26.1×
TbTc4	52.8	2.3	2.1×	2.9×	23.0×
TbTc5	40.3	1.8	5.9×	6.1×	22.4×
TbTc6	49.0	2.3	3.5×	6.0×	21.3×
TbN1	38.7	1.6	15.5×	4.6×	24.2×
TbN2	40.4	5.9	2.3×	1.4×	6.8×
TbN3	33.2	2.5	9.8×	1.4×	13.3×
TbN4	40.5	3.8	7.1×	1.5×	10.7×

a Relative GFP fluorescence of TOBRA hybrid riboswitches in the absence (wo) and presence of 250 μM of both of their respective ligands. A background was subtracted (pCBB06) and GFP expression was normalized to mCherry expression. The positive control under the same ligand condition as each measurement was set to 100%.

TbN1 (N4D9) was the best performing TOBRA-NEO candidate with a 24.2-fold dual-input dynamic range and 38.7% basal expression (Table 4). The connecting sequence of TbN1 occurred in 50% of all candidates remaining after the screening process. High enrichment of a sequence after sorting was expected due to the low initial variability of the TOBRA-NEO N4 pool (256 variants possible). TbN1 achieved a 15.5-fold dynamic range in the presence of 250 μM tobramycin, making it the hybrid riboswitch with the highest single-input dynamic range. The basal expression and tobramycin single-input dynamic range of TbN1 even surpasses the parental TOBRA N6G5 riboswitch. The 4.6-fold dynamic range in the presence of 250 μM neomycin was also the highest measured for a TOBRA-NEO construct, with all other candidates conveying regulation mostly through the binding of tobramycin (Figure 5B).

## 3 Discussion

In this study multiple dual-input riboswitches outperforming their parental single-input riboswitches in both dynamic range and basal expression were created (PTc4, PTb5, TN3, TcTb5/6, TbTc1, TbN1). Constructs created from the strongest parental riboswitches, TC and TOBRA, showed the highest performance. When choosing the best performing hybrid construct, it is important to consider that both basal expression and dynamic range play a role in the overall performance of a riboswitch. If a balanced combination of both factors is desired, the TC-TOBRA hybrid TcTb5 with up to 68% basal expression and an 18.8-fold dual-input dynamic range can be considered the overall best performing riboswitch for the roadblock mechanism in yeast to date.^14^ If dynamic range is considered the more critical factor, the rationally designed construct TcTb6 achieves values comparable to those recorded for a TC-Dimer optimized via machine learning (TcTb6:36.1×; TC-Dimer: 40×), while reaching a higher basal expression (TcTb6: 46%; TC-Dimer: 25%).^23^ In the case of TC-NEO hybrids a comparison can also be made with a published dimer utilizing the full sequences of both parental riboswitches directly adjacent to each other.^25^ For this tandem construct NEO was placed upstream of TC, with a 30 nucleotide long single-stranded linker separating both riboswitches. While the strongest TC-NEO hybrid TN3 outperforms this tandem riboswitch in both dynamic range and basal expression, a direct comparison is difficult due to the stronger TC G6 variant not being available for the tandem riboswitch design at the time of construction.^23^ It is however important to note that the hybrid achieves this performance while being significantly shorter in total sequence length (NEO-TC tandem: 132 nt; TN3 hybrid: 78 nt). Constructs presented in this study have made a significant improvement to the overall sequence length required for a functional dual-input riboswitch.

While it is difficult to name general guidelines for the creation of hybrid riboswitches, due to the individual regulation mechanisms and scaffolds of each parental candidate, our results suggest that simple rules of rational design (truncations, stem variations, loop exchanges) can be used in most cases. We have found that scaffold sequence variants (e.g., TC P1 stems; TOBRA loop) have comparable effects in both parental and hybrid riboswitches. Utilizing bulges as part of the connecting stem sequence can also have a positive effect on the resulting hybrid, especially when dealing with highly structured parental riboswitches (TC-TOBRA; PARO-TOBRA). When designing a sequence, we advise to avoid the occurrence of premature start codons, as these can lead to reduced expression of the primary open reading frame. When choosing a design strategy, we propose that structure-based design approaches (artificial linker: PARO-TOBRA; TC-TOBRA) favor rational design over randomized screening. This is because long randomized sequences are difficult to cover in a screening approach without missing potentially viable candidates due to the large amount of variability. Additionally, the introduction of a structure element (bulge) at a specific position in a stem can be tested in less time when utilizing single-sequence measurements. When focusing on short, direct connections via a stem (PARO-TC; TOBRA Hybrids), predicting the outcome of sequence variations is difficult. A randomized-screening approach is therefore favorable. Both structure-based rational design and a sequence-based randomized screening resulted in viable and highly efficient hybrid riboswitches capable of outperforming their parental riboswitches.

It is important to note that the concept of fusing two aptamer domains has been used previously for the design of biosensors. Fluorogenic aptamers like Spinach have been fused to metabolite sensing aptamer domains for visualization and quantification of their respective ligands in living cells.^32−34^ These fusions add more functionality to ligand-sensing aptamer domains, but unlike hybrid riboswitches, provide no improvement to potentially existing regulatory functions. Besides rational design, several types of dual-input tandem riboswitches have already been discovered in nature. The first reported construct capable of binding two different ligands was the SAM-AdoCbl tandem riboswitch discovered in *B. clausii*.^24^ During later studies different types of dual-input riboswitches were discovered, among them also candidates utilizing distinct binding pockets to influence the same expression platform, an intrinsic terminator stem, similar in concept to the synthetic hybrid riboswitches.^35,36^ This tandem construct containing aptamers for both 5-phosphoribosyl-1-pyrophosphate (PRPP) and guanine presented an interesting switching pattern, in which the binding of PRPP stimulated gene expression, whereas the presence of guanine had the opposite effect.^35^ A conclusion drawn from this was that constructs containing binding pockets capable of directly influencing each other may be beneficial for the creation of a broader collection of logical functions.^36^ Dual-input hybrid riboswitches have a similar architecture, that also uses two distinct binding pockets to influence the same expression platform (in their case overall construct stability). It therefore stands to reason that hybrid constructs may be a beneficial choice for the design of genetic regulators capable of emulating various Boolean logic gates.

With this study we showed that dual-input regulation of the riboswitch roadblock translation control mechanism in yeast can easily be achieved by combining the binding pockets of different riboswitches into one compact structure. We combined several well-researched parental riboswitch candidates in all functional orientations using different design strategies to show the simplicity of creating hybrid riboswitches. From our results we hypothesize that potentially all currently available riboswitches capable of the roadblock mechanism in yeast can be used for the design of hybrid riboswitches.

Due to the high dynamic range and basal expression levels, the compact size and the low metabolic burden, utilizing hybrid riboswitches will be a beneficial choice for the development of biotechnological applications. Their dual-input nature, combined with the close proximity of both binding pockets within most hybrid riboswitches additionally increases the chance of a construct to achieve cooperative binding. This aspect of hybrid riboswitches will make them interesting targets for future projects focused on engineering their switching patterns to create various types of synthetic Boolean logic gates for the design of complex genetic circuits. However, developing such a hybrid riboswitch will require more sophisticated design approaches, most likely a screening combined with machine learning to predict the sequence–structure relationship necessary to emulate a Boolean logic gate.

## 4 Methods

### 4.1 Plasmid Construction

All oligonucleotides used in this study were purchased from Sigma-Aldrich and are listed in Table S2. All constructs were cloned using the vector pCBB06, a variant of the plasmid pCBB05.^16^ pCBB05 contains a *gfp* gene driven by an ADH1 promotor and an *mCherry* gene controlled by a TEF promotor that is used for normalization of GFP expression. An AgeI and an NheI restriction site are located immediately up- and downstream of the *gfp* start codon. The start codon and adjacent Kozak sequence of the *gfp* gene were replaced by a CTCTTC sequence in plasmid pCBB06 to enable GFP expression only after correct insertion of an insert containing a riboswitch sequence and the start codon. Inserts were generated through hybridization of oligonucleotides with overhangs corresponding to the restriction sites of AgeI and NheI. Constructs were assembled through ligation of the inset with pCBB06 linearized by AgeI-HF and NheI-HF (NEB). *Escherichia coli* TOP10 (Invitrogen) were transformed with the ligation products. Plasmid extraction from overnight cultures inoculated with single colonies was performed using the PureYield Plasmid Miniprep System (Promega). Sequences were verified via Sanger Sequencing (Microsynth Seqlab).

### 4.2 Yeast Cultivation and GFP Measurements

*Saccharomyces cerevisiae* RS453α cells (MATα *ADE*2-1 *TRP*1-1 *CAN*1-100 *LEU*2-3 *HIS*3-1 *URA*3-52) were transformed with verified plasmid DNA using the Frozen-EZ Yeast Transformation II Kit (Zymo Research). Transformed cells were plated on SCD-ura plates [0.2% YNB w/o AA (Difco), 0.55% ammonium sulfate (Roth), 2% glucose (Roth), 12 μg/mL adenine (SIGMA), 1× MEM amino acids (SIGMA), 2% agar (Oxoid)] and incubated in a humidified incubator at 30 °C for 48 h. SCD-Ura cultures (1.5 mL) were inoculated with the yeast colonies of each candidate and incubated for 24 h in 24-well plates at 30 °C and 450 rpm. Cultures were diluted 1:1000 after incubation in fresh media containing either no ligand, 250 μM of one ligand, or 250 μM of both ligands. After another 24 h of incubation 20 μL of culture were transferred into 180 μL fresh SCD-Ura in a 96-well plate. The fluorescence of GFP and mCherry was measured for 25,000 cells per candidate using the CytoFlex S cytometer (Beckman Coulter) (GFP: 488 nm laser (bandpass filter: 510/520 nm); mCherry: 561 nm laser (bandpass filter: 610/620 nm)). A positive (pCBB05) and negative (pCBB06) control were measured in parallel. The GFP fluorescence of each candidate and control was normalized to its respective mCherry fluorescence and the values of the negative control were subtracted from all candidates and the positive control. The adjusted value of each sample was normalized to the positive control incubated with the equivalent ligand condition. A biological duplicate was measured for every candidate and every experiment was performed in technical triplicates at independent days.

### 4.3 Homologous Recombination of Yeast Libraries

A pool of hybrid riboswitch sequences with randomized regions was amplified using the Q5 High-Fidelity DNA polymerase (NEB) according to the supplieŕs instructions to add sequences homologous to regions up- and downstream of the *gfp* start codon on the vector pCBB06. The PCR product was purified using the Wizard SV Gel and PCR Clean-Up System (Promega). For yeast transformation 4.5 μg of purified PCR product were mixed with 1.5 μg of double-digested (AgeI-HF and NheI-HF) and purified vector pCBB06. Yeast cells were transformed by electroporation.^37^ Two transformations were performed for each pool to increase transformation efficiency and the transformed cells were combined in 250 mL SCD-Ura in a 2 L culture flask and incubated for 48 h at 30 °C and 110 rpm. Serial dilutions on SCD-Ura agar plates were performed and the plates were incubated for 72 h at 30 °C in a humidified incubator to determine transformation efficiency. Serial dilution steps of the liquid culture in fresh SCD-Ura (250 mL) were performed according to the electroporation protocol after the initial 48 h incubation to avoid the occurrence of cells containing more than one plasmid (1/10, 1/30, 1/100; 24 h incubation after each dilution).

### 4.4 Fluorescence-Based Cell Sorting and Screening

Hybrid riboswitch yeast library pools and controls were used to inoculate (1:100) 10 mL SCD-Ura cultures (100 mL culture flasks) containing either no ligand or 250 μM of both ligands (tobramycin and tetracycline/neomycin) and incubated for 24 h at 30 °C and 130 rpm in a humidified incubator. Positive (pCBB05) and negative (pCBB06) controls were created by transforming yeast cells using the Frozen-EZ Yeast Transformation II Kit (Zymo Research). For cell sorting cultures were diluted 1:10 in 10 mL fresh SCD-Ura media. The CytoFlex SRT cell sorter (Beckman Coulter) [488 nm laser (GFP); 561 nm laser (mCherry)] was used for the sorting process. Emission light was bandpass filtered at 525/540 nm and 610/620 nm. Initial sorting parameters were set to exclude cells of irregular shape, doublets (connected cells) and cells without constitutive mCherry expression. GFP was normalized to mCherry expression and the controls were used to determine the sorting range (0–100% normalized expression, Figure S9). In the first sorting 10,000 cells (N4 or N6 pools) or 100,000 cells (N8 pool) with 10% or less normalized fluorescence in the presence of both ligands were sorted into 5 mL fresh SCD-Ura. The number of sorted events for each pool was based on the amount of events discovered in the desired sorting range (roughly 10% of all events exhibited fluorescence values between 0 and 10%). Sorted cultures were incubated for 48 h at 30 °C and 140 rpm in a humidified incubator and afterward used to inoculate new 10 mL SCD-Ura cultures containing either no ligand or 250 μM of both ligands. After a 24 h incubation period at 30 °C and 130 rpm cultures were again diluted (1:10 in 10 mL fresh SCD-Ura) and 10,000 cells were sorted from the 5% of the population achieving the highest normalized fluorescence in the absence of a ligand. The entire sorting process (first and second sorting) was repeated using the same parameters to further enrich candidates with a high dynamic range (Figure S10). For the screening process single cells of the enriched population were used to inoculate a 96-deep-well plate containing 300 μL SCD-Ura per well and incubated for 48 h at 30 °C and 1000 rpm in a plate incubator. Two 96-deep-well plates containing 300 μL SCD-Ura with no ligand or 250 μM of both ligands per well were prepared and inoculated from the incubated plate. After a 24 h incubation period at 30 °C and 1000 rpm 25,000 cells of each well from both plates were measured using the CytoFlex S cytometer (Beckman Coulter, device specifications see above). GFP was normalized to mCherry expression and the results from each candidate in the absence and presence of both ligands were used to pick candidates with a potential for a high dynamic range. Plasmid extraction from chosen candidates was performed using the PureYield Plasmid Miniprep System (Promega). During plasmid extraction an additional step was taken after addition of the lysis buffer: glass sand was added to the samples and a FastPrep-24 homogenizer (MP Biomedicals) was used for two 30 s intervals at 6.5 m/s to rupture the cell walls. The supernatant was transferred into a new tube after debris and glass sand had settled and the protocol was continued according to the supplier’s instructions. After a passage through *E. coli* candidate sequences were determined using Sanger sequencing (Microsynth Seqlab) and used for retransformation of yeast cells to conduct GFP measurements (see Yeast Cultivation and GFP Measurements).

## Supporting Information Available

Measurements of all parental riboswitch variants (Figure S1), linker sequence truncations of PARO variants (Figure S2), ligand effects on GFP and mCherry fluorescence (Figure S3), cross-reactivity assays of parental riboswitches under all double-ligand conditions (Figure S4), PARO-NEO hybrid riboswitch variants with a direct connection (Figure S5), TC-NEO hybrid riboswitches created using NEO variant M7 (Figure S6) and TC-TOBRA hybrid riboswitches with a direct connection (Figure S7). A schematic representation of the screening for TOBRA hybrid riboswitches (Figure S8), the initial sorting parameters (Figure S9), and the individual gates used for each pool and sorting step are also shown (Figure S10). Table S1 provides information on yeast growth times under all double-ligand conditions. Table S2 provides the sequences of all constructs shown in this study. Table S3 provides information on dynamic ranges and expression values of all constructs (PDF)
