# Supplementary material for: Synthetic Dual-Input Hybrid Riboswitches—Optimized Genetic Regulators in Yeast
Source: ACS Synth Biol. 2025 Feb 4;14(2):497–509. doi: 10.1021/acssynbio.4c00660 (PMC11854369; doi:10.1021/acssynbio.4c00660)
Supplement: Supplementary file 1 — sb4c00660_si_001.pdf [file sb4c00660_si_001.pdf]

Supplementary Information

Synthetic dual-input hybrid riboswitches – optimized genetic regulators in yeast

Daniel Kelvin[1], Janette Arias Rodriguez[1], Ann-Christin Groher[1], Kiara Petras[1] and Beatrix Suess[1,2]*

[1] Fachbereich Biologie, TU Darmstadt, Schnittspahnstrasse 10, 64287 Darmstadt, Germany
[2] Centre for Synthetic Biology, TU Darmstadt, 64287 Darmstadt, Germany

* to whom correspondence should be addressed: bsuess@bio.tu-darmstadt.de

Contents

Figure S1 – Parental riboswitch variants **2**

Figure S2 – PARO linker modifications **4**

Figure S3 – Ligand effects on GFP and mCherry **5**

Figure S4 – Parental riboswitch cross reactivity assay **6**

Figure S5 – PARO-NEO hybrids with direct connection **7**

Figure S6 – TC-NEO M7 hybrids **8**

Figure S7 – TC-TOBRA hybrids with direct connection **9**

Figure S8 – FACS workflow **10**

Figure S9 – TOBRA screening I **11**

Figure S10 – TOBRA Screening II **12**

Supplementary references **13**

Table S1 – Yeast generation time under double ligand conditions **14**

Table S2 – Oligonucleotide sequences **15**

Table S3 – Supplementary hybrid constructs dynamic ranges **18**

Figure S1 – Parental riboswitch variants

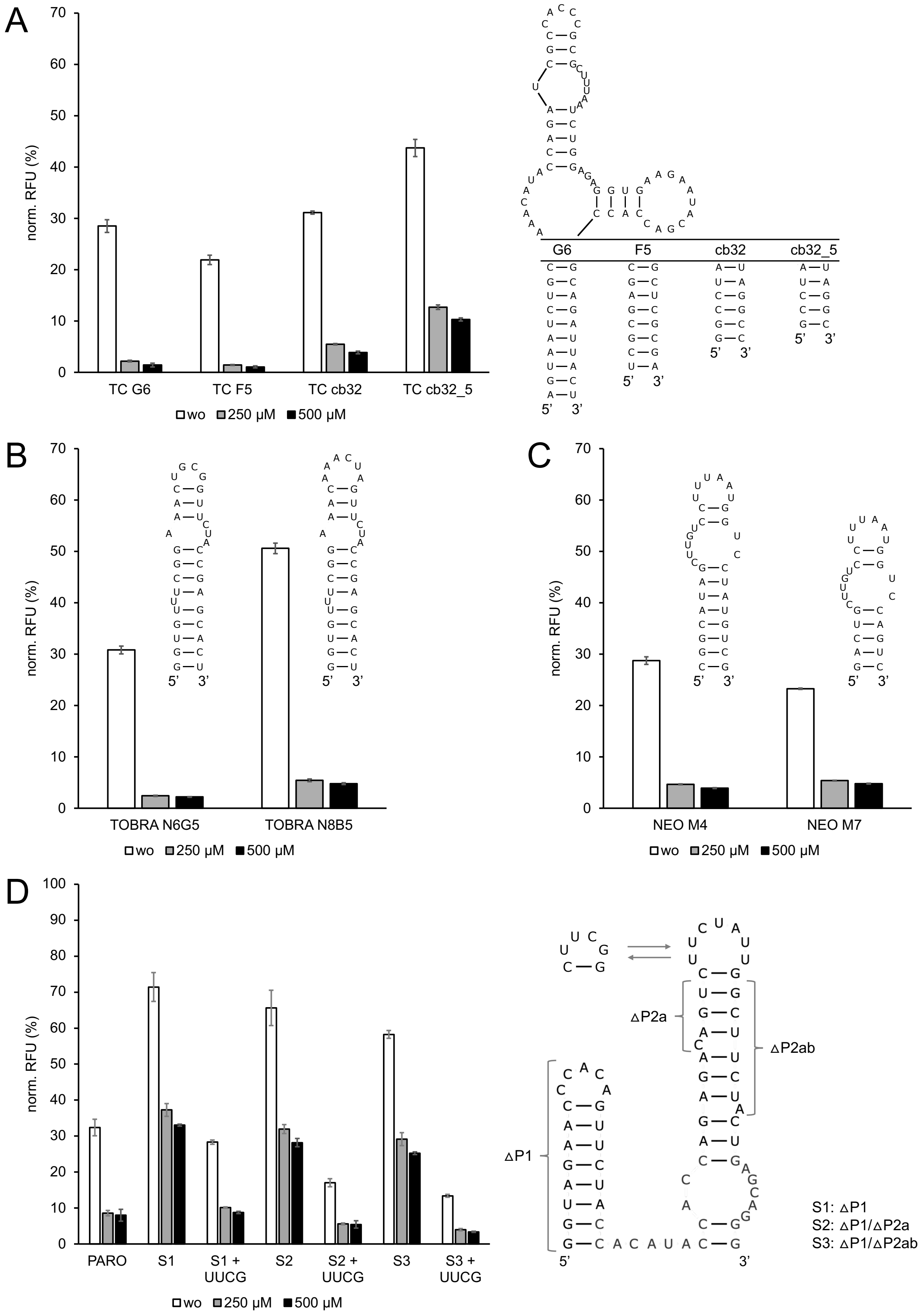

Figure S1. Measurements of the influence of different parental riboswitch variants on GFP expression in the absence (wo; white bars) or presence of their respective ligands (250 µM (grey bars) and 500 µM (black bars) ligand concentration). (A) TC P1 stem variants G6, F5, cb32 and cb32_5[1,2]. (B) TOBRA terminal loop variants N6G5 and

N8B5[3]. (C) NEO stem variants M4 and M7[4]. (D) Truncations of PARO[5]. The P1 stem was removed for variants S1-3. The P2 stem has also been truncated to a varying degree in the constructs S2 and S3. The terminal loop of P2 was also exchanged for a more stable UUCG loop in all truncated versions of PARO to test its effect on expression levels. *S. cerevisiae* RS453 were transformed with the plasmid pCBB06[5] containing the respective riboswitch in the 5`UTR of *gfp* directly upstream of the start codon. GFP fluorescence was normalized to the fluorescence of constitutively expressed mCherry. pCBB06 without a riboswitch served as a blank and its GFP fluorescence was subtracted from all other measurements. The positive control under the same ligand condition as each measurement was set to 100%. Measurements were performed in duplicates and repeated twice. Switching efficiencies can be viewed in Table 1.

Figure S2 – PARO linker modifications

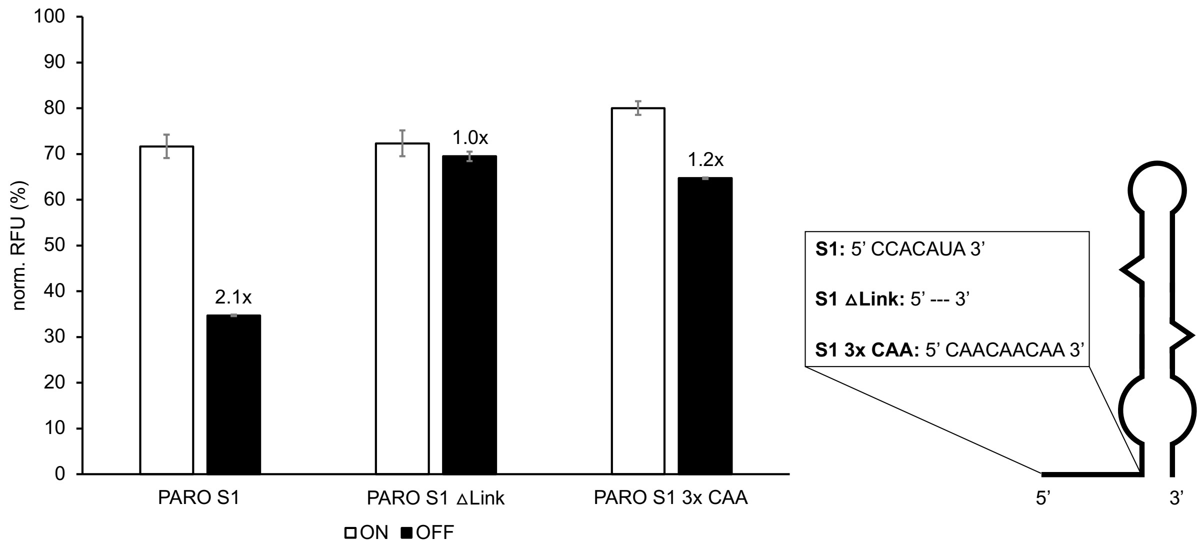

Figure S2. The single-stranded linker sequence of PARO S1[5], which can be viewed in Figure S1D, was removed (∆Link) or exchanged for a generic $(CAA)_3$ sequence to test its effect on riboswitch performance. For all measurements *S. cerevisiae* RS453 were transformed with the plasmid pCBB06[5] containing the respective hybrid riboswitch in the 5`UTR of *gfp* directly upstream of the start codon. GFP fluorescence was normalized to the fluorescence of constitutively expressed mCherry. pCBB06 without a riboswitch served as a blank and its GFP fluorescence was subtracted from all other measurements. The positive control under the same ligand condition as each measurement was set to 100%. Measurements were performed in duplicates and repeated twice.

Figure S3 – Ligand effects on GFP and mCherry

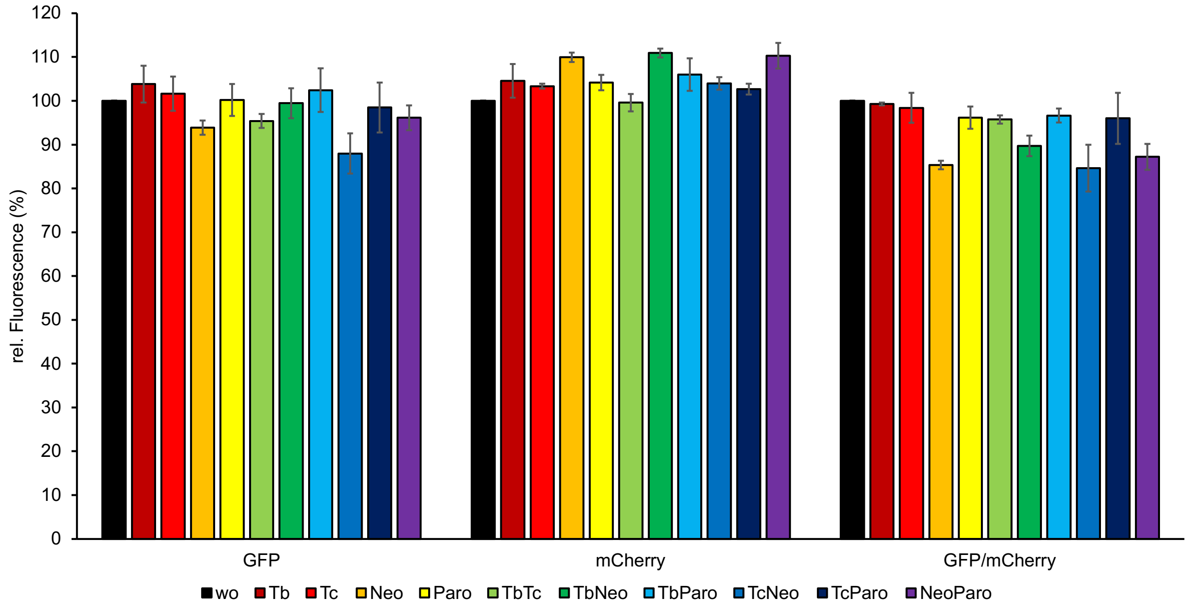

Figure S3. Ligand effects on measured fluorescence values. Effects of both the individual (250 µM) and double ligand conditions (250 µM of each ligand) on GFP and mCherry fluorescence values were determined using the positive control containing no riboswitch sequence (*S. cerevisiae* RS453 were transformed with the plasmid pCBB05). For both GFP and mCherry the measurement in the absence of a ligand (wo) was set to 100%. pCBB06 served as the blank and its GFP fluorescence was subtracted from all other results. GFP values were normalized to mCherry to visualize the combined ligand effect. Measurements were performed in duplicates and repeated twice.

Figure S4 – Parental riboswitch cross reactivity assay

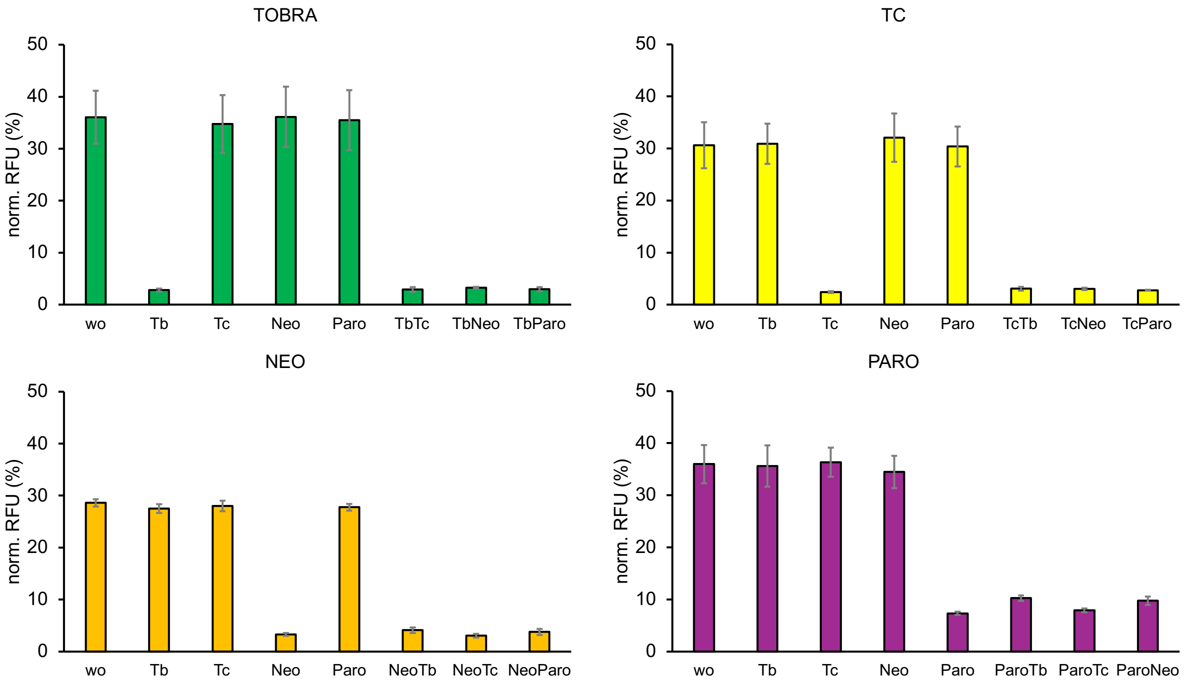

Figure S4. Assay for parental riboswitch ligand specificity under both single- and double-ligand conditions. The influence on gene expression of parental riboswitch variants TOBRA N6G5[3] (upper left), TC G6[2] (upper right), NEO M4[4] (lower left) and PARO[5] (lower right) was measured after a 24 h incubation period without a ligand (wo), or with 250 µM tobramycin (Tb), tetracycline (Tc), neomycin (Neo), paromomycin (Paro). All double-ligand conditions (2x 250 µM) of hybrids originating from the parental riboswitches were also tested for the respective constructs (e.g. both tobramycin (250 µM) and tetracycline (250 µM), neomycin (250 µM) or paromomycin (250 µM) for TOBRA N6G5: TbTc, TbNeo, TbParo). For all measurements *S. cerevisiae* RS453 were transformed with the plasmid pCBB06[5] containing the respective hybrid riboswitch in the 5`UTR of *gfp* directly upstream of the start codon. GFP fluorescence was normalized to the fluorescence of constitutively expressed mCherry. pCBB06 without a riboswitch served as a blank and its GFP fluorescence was subtracted from all other measurements. The positive control under the same ligand condition as each measurement was set to 100%. Measurements were performed in duplicates and repeated twice.

Figure S5 – PARO-NEO hybrids with direct connection

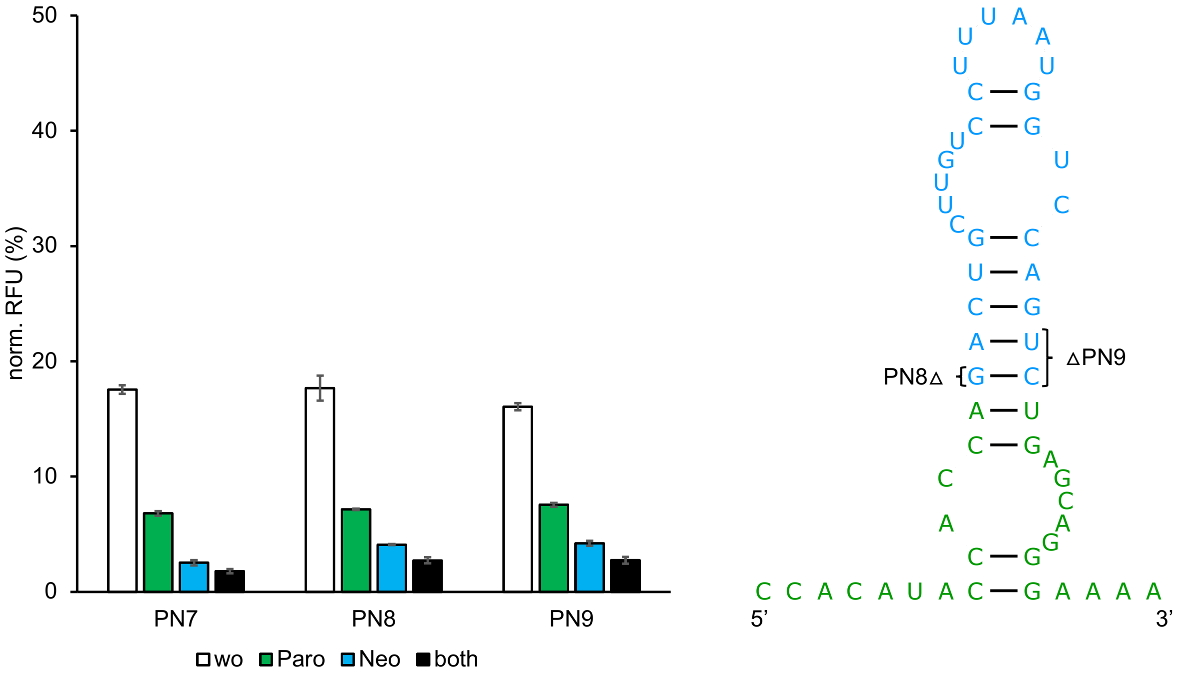

Figure S5. GFP measurements of directly connected PARO-NEO[4,5] hybrid riboswitches. Candidates were measured in the absence (wo) and presence of 250 µM paromomycin (Paro) and 250 µM neomycin (Neo) individually and simultaneously (both). The 2D structure of PN7 is shown, with sequences originating from PARO being marked in green and sequences originating from NEO M7 being marked in blue. For all measurements *S. cerevisiae* RS453 were transformed with the plasmid pCBB06[5] containing the respective hybrid riboswitch in the 5`UTR of *gfp* directly upstream of the start codon. GFP fluorescence was normalized to the fluorescence of constitutively expressed mCherry. pCBB06 without a riboswitch served as a blank and its GFP fluorescence was subtracted from all other measurements. The positive control under the same ligand condition as each measurement was set to 100%. Measurements were performed in duplicates and repeated twice. Switching efficiencies can be viewed in Table S3.

Figure S6 – TC-NEO M7 hybrids

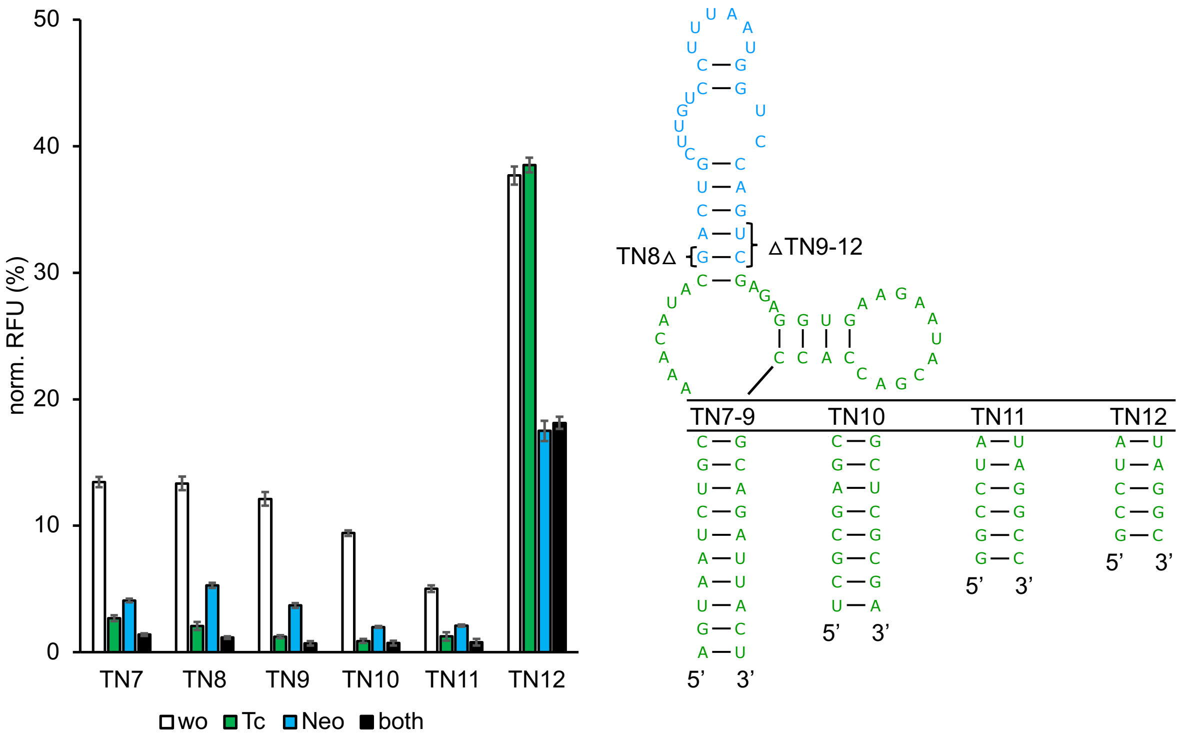

Figure S6. GFP measurements of TC-NEO[1,2,4] hybrid riboswitches created using the NEO stem variant M7. Constructs were measured in the absence (wo) and presence of 250 µM tetracycline (Tc) and 250 µM neomycin (Neo) individually and simultaneously (both). The 2D structure of TN7 is shown, with sequences originating from TC being marked in green and sequences originating from NEO M7 being marked in blue. Truncations of the connecting stem corresponding to other TC-NEO constructs, as well as the TC P1 stem variants used by each construct (TN7-9: TC G6; TN10: TC F5; TN11: TC cb32; TN12: Tc cb32_5) are shown. For all measurements *S. cerevisiae* RS453 were transformed with the plasmid pCBB06[5] containing the respective hybrid riboswitch in the 5`UTR of *gfp* directly upstream of the start codon. GFP fluorescence was normalized to the fluorescence of constitutively expressed mCherry. pCBB06 without a riboswitch served as a blank and its GFP fluorescence was subtracted from all other measurements. The positive control under the same ligand condition as each measurement was set to 100%. Measurements were performed in duplicates and repeated twice. Switching efficiencies can be viewed in Table S3.

Figure S7 – TC-TOBRA hybrids with direct connection

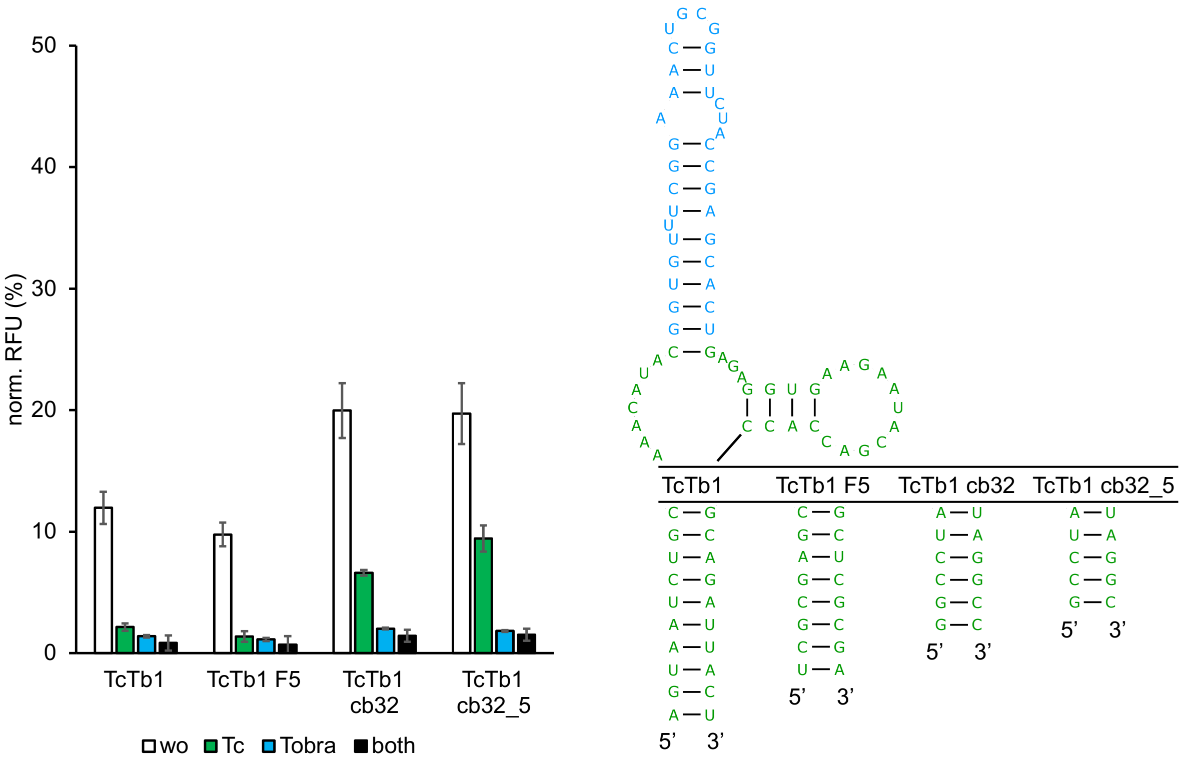

Figure S7. GFP measurements of directly connected TC-TOBRA[1–3] hybrid riboswitches. Candidates were measured in the absence (wo) and presence of 250 µM tetracycline (Tc) and 250 µM tobramycin (Tb) individually and simultaneously (both). The 2D structure of TcTb1 is shown, with sequences originating from TC being marked in green and sequences originating from TOBRA N6G5 being marked in blue. TC P1 stem variants used by each construct (TcTb1: TC G6; TcTb1 F5: TC F5; TcTb1 cb32: TC cb32; TcTb1 cb32_5: Tc cb32_5) are shown. For all measurements *S. cerevisiae* RS453 were transformed with the plasmid pCBB06[5] containing the respective hybrid riboswitch in the 5`UTR of *gfp* directly upstream of the start codon. GFP fluorescence was normalized to the fluorescence of constitutively expressed mCherry. pCBB06 without a riboswitch served as a blank and its GFP fluorescence was subtracted from all other measurements. The positive control under the same ligand condition as each measurement was set to 100%. Measurements were performed in duplicates and repeated twice. Switching efficiencies can be viewed in Table S3.

Figure S8 – FACS workflow

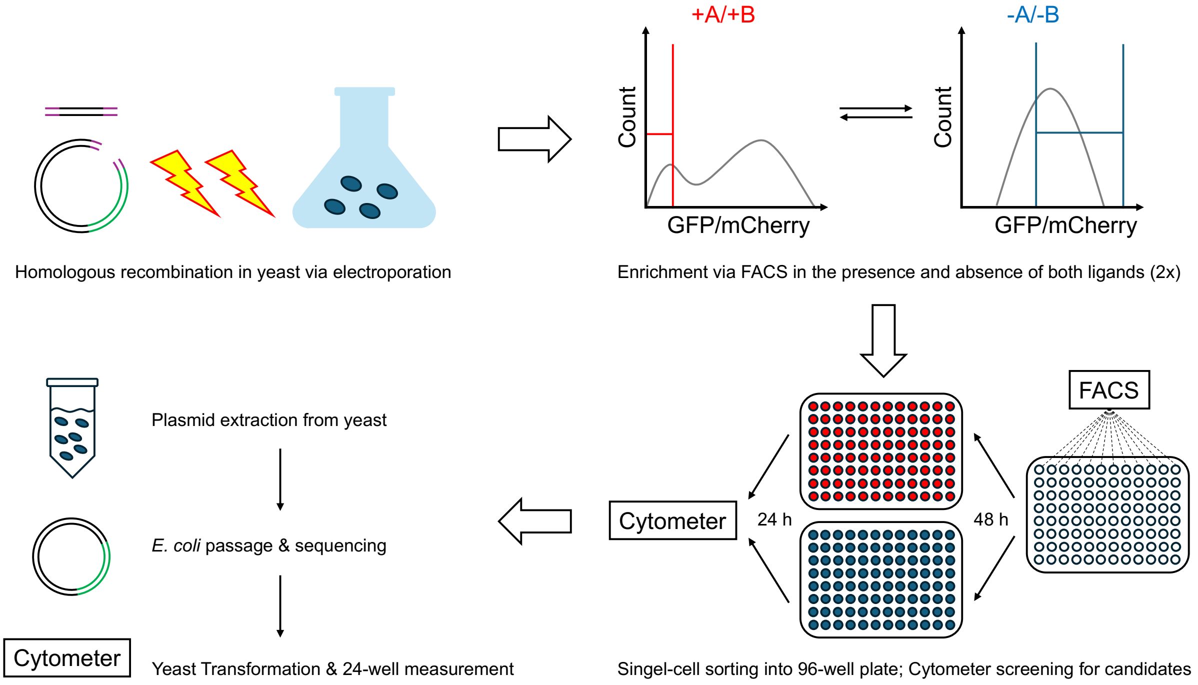

Figure S8. Workflow of screening process for randomized TOBRA hybrid pools. *S. cerevisiae* RS453 cells were transformed with the linearized vector pCBB06[5] (double-digested by AgeI-HF and NheI-HF) and TOBRA hybrid library sequences containing overhangs added via PCR, that are homologus to the ends of the vector. Yeast cells were transformed using a high-efficiency electroporation protocol to generate yeast libraries capable of covering all potential combinations (transformation efficiency > 106 cfu/µg)[6]. Plasmids were created through homologous recombination by the yeast cells. Fluorescence-based cell sorting was used to enrich functional candidates with high dual-input dynamic range in each pool. GFP fluorescence was normalized to mCherry fluorescence for the entire screening process. The pools were initially sorted for low fluorescence in the presence of both ligands (+A/+B). A second sorting step was used to select for candidates with high fluorescence in the absence of both ligands (-A/-B) from the enriched subpopulation of the first round. The entire process was repeated to further enrich desired candidates and remove false-positives. Potential candidates were separated into a 96-well plate (one plate per pool). After the single-cell sorting a 48 h incubation period was used to increase cell density before using the initial 96-well plates to inoculate two new 96-well plates (1:100 dilution in new plates). The first plate contained SCD-Ura medium with 250 µM of both ligands (tobramycin and tetracycline or neomycin; red), while the second plate contained SCD-Ura medium without a ligand (blue). The plates were measured after a 24 h incubation period for GFP and mCherry fluorescence and the data for each candidate between both plates was compared to select hybrids with high dynamic range. Overnight cultures of the selected candidates were inoculated and used for plasmid extraction. Transformation of *E. coli* was performed to increase plasmid yield and after another plasmid extraction DNA sequences were verified using Sanger sequencing. *S. cerevisiae* RS453 were transformed with the plasmids and fluorescence values for GFP and mCherry were measured for all ligand conditions in a 24-well plate. GFP fluorescence was normalized to the fluorescence of constitutively expressed mCherry. pCBB06 without a riboswitch served as a blank and its GFP fluorescence was subtracted from all other measurements. The positive control under the same ligand condition as each measurement was set to 100%. Measurements were performed in duplicates and repeated twice.

Figure S9 – TOBRA screening I

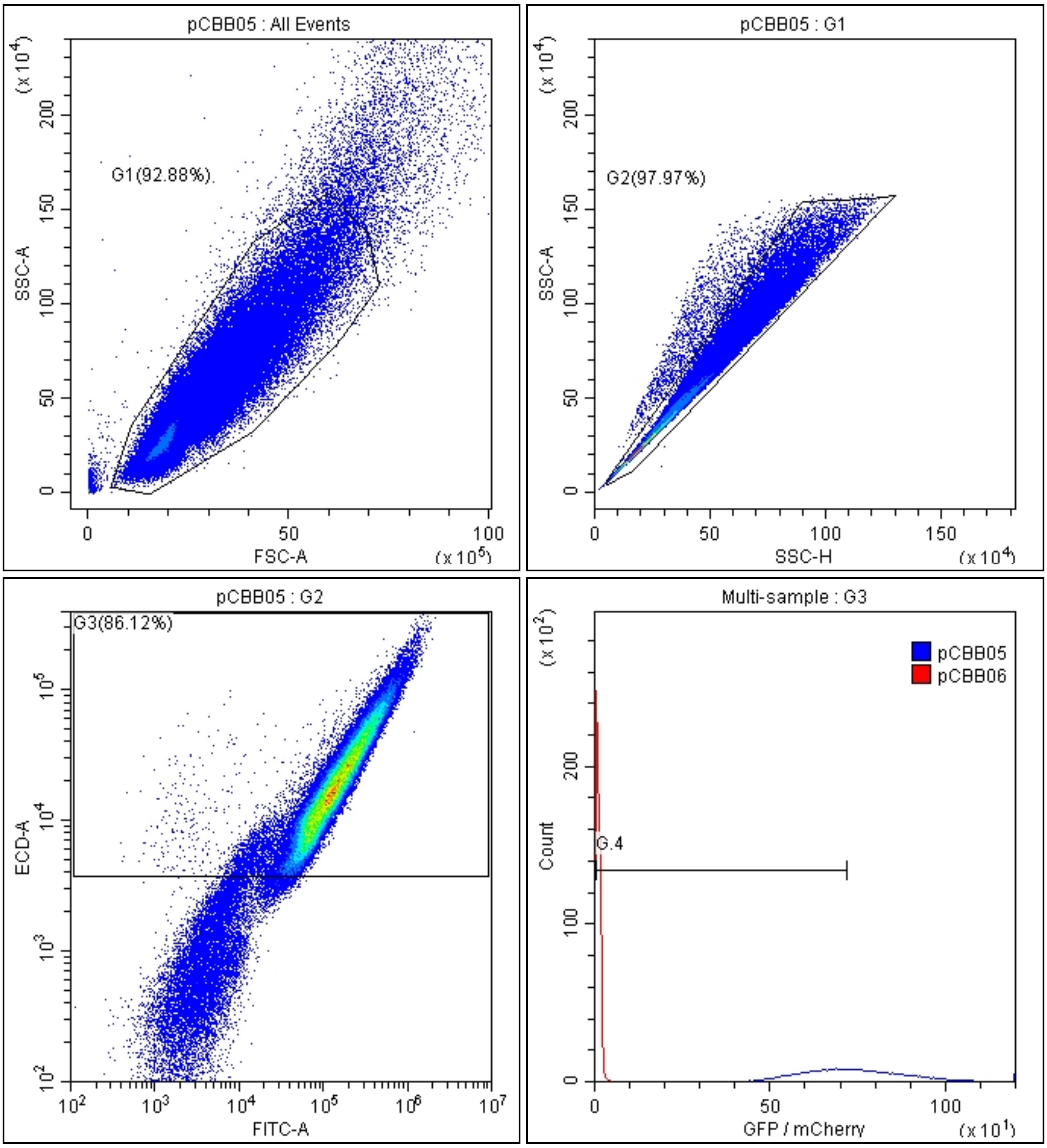

Figure S9. Initial gates for TOBRA screening. Initial sorting parameters were set to exclude cells of irregular shape (G1), doublets (connected cells)(G2) and cells without constitutive mCherry expression (G3; ECD: mCherry; FITC: GFP). GFP was normalized to mCherry expression and the controls without a riboswitch (pCBB05: positive; pCBB06: negative (no GFP start codon)) were used to determine the sorting range (0-100% normalized expression)(G4).

Figure S10 – TOBRA Screening II

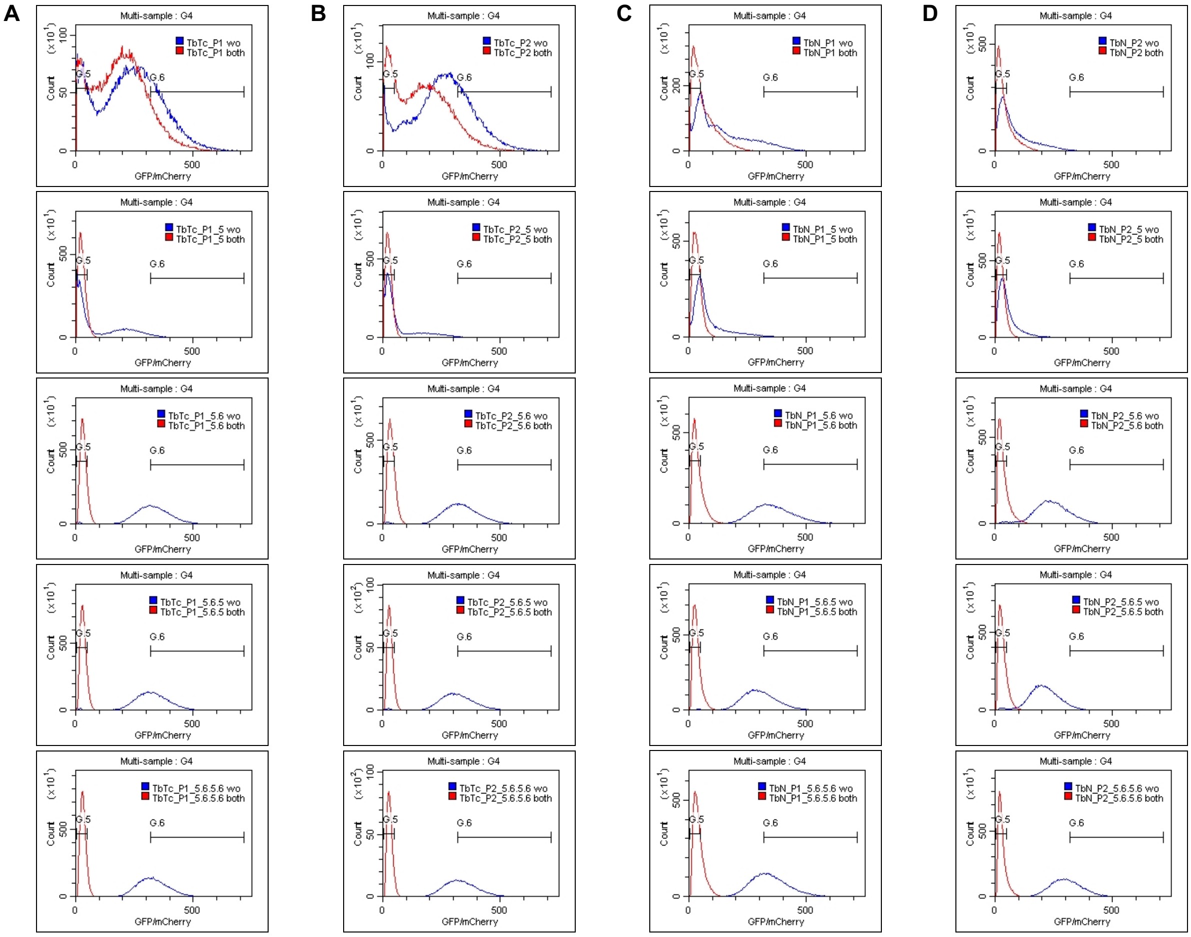

Figure S10. Sorting rounds for all TOBRA pools. The four different TOBRA pools (A) TOBRA-TC P1(N6), (B) TOBRA-TC P2(N8), (C) TOBRA-NEO P1(N4) and (D) TOBRA-NEO P2(N6) were first sorted for low fluorescence (Gate G5) in the presence of both ligands (both), and afterwards for high fluorescence (Gate G6) in the absence of a ligand (wo). The entire sorting process was repeated to further enrich candidates with high dynamic ranges.

Supplementary references

(1) Suess, B.; Hanson, S.; Berens, C.; Fink, B.; Schroeder, R.; Hillen, W. Conditional Gene Expression by Controlling Translation with Tetracycline-Binding Aptamers. *Nucleic Acids Res* **2003**, *31* (7), 1853–1858. https://doi.org/10.1093/nar/gkg285.

(2) Groher, A. C.; Jager, S.; Schneider, C.; Groher, F.; Hamacher, K.; Suess, B. Tuning the Performance of Synthetic Riboswitches Using Machine Learning. *ACS Synth Biol* **2019**, *8* (1), 34–44. https://doi.org/10.1021/acssynbio.8b00207.

(3) Kraus, L.; Duchardt-Ferner, E.; Bräuchle, E.; Fürbacher, S.; Kelvin, D.; Marx, H.; Boussebayle, A.; Maurer, L. M.; Bofill-Bosch, C.; Wöhnert, J.; Suess, B. Development of a Novel Tobramycin Dependent Riboswitch. *Nucleic Acids Res* **2023**, *51* (20), 11375–11385. https://doi.org/10.1093/nar/gkad767.

(4) Weigand, J. E.; Sanchez, M.; Gunnesch, E. B.; Zeiher, S.; Schroeder, R.; Suess, B. Screening for Engineered Neomycin Riboswitches That Control Translation Initiation. *RNA* **2008**, *14* (1), 89–97. https://doi.org/10.1261/rna.772408.

(5) Boussebayle, A.; Torka, D.; Ollivaud, S.; Braun, J.; Bofill-Bosch, C.; Dombrowski, M.; Groher, F.; Hamacher, K.; Suess, B. Next-Level Riboswitch Development-Implementation of Capture-SELEX Facilitates Identification of a New Synthetic Riboswitch. *Nucleic Acids Res* **2019**, *47* (9), 4883–4895. https://doi.org/10.1093/nar/gkz216.

(6) Benatuil, L.; Perez, J. M.; Belk, J.; Hsieh, C. M. An Improved Yeast Transformation Method for the Generation of Very Large Human Antibody Libraries. *Protein Engineering, Design and Selection* **2010**, *23* (4), 155–159. https://doi.org/10.1093/protein/gzq002.

Table S1 – Yeast generation time under double ligand conditions

**Condition***	**Generation time**
No ligand	69.9 ± 3.2 min
250 µM paromomycin<br>250 µM tetracycline	73.3 ± 3.5 min
250 µM paromomycin<br>250 µM tobramycin	69.3 ± 4.1 min
250 µM paromomycin<br>250 µM neomycin	72.1 ± 2.4 min
250 µM tetracycline<br>250 µM neomycin	77.8 ± 5.5 min
250 µM tetracycline<br>250 µM tobramycin	71.3 ± 2.5 min
250 µM tobramycin<br>250 µM neomycin	68.8 ± 6.5 min

* measured in a 24-well plate in triplicates (3x 1.5 ml SCD-Ura medium per condition). Inoculation 1:1000 with RS453 yeast cells transformed with the plasmid pCBB05 and incubated in SCD-Ura medium for 24 h in a plate incubator at 30 °C (shaking). $OD_{600\ nm}$ measured in 15 min intervals at 30°C (shaking) for 24 h using a TECAN Infinite M200 Pro plate reader. Wells containing medium without cells were used as the blank. The experiment was repeated twice.

Table S2 – Oligonucleotide sequences

Construct	Sequence (5' → 3')
TC G6	cagtaatctgcaaacataccagatcgccacccgcgctttaatctggagaggtgaagaatacgaccacc gcagattact
TC F5	ctcgcgagcaaacataccagatcgccacccgcgctttaatctggagaggtgaagaatacgaccacc gctcgcga
TC cb32	cggcctaaaacataccagatcgccacccgcgctttaatctggagaggtgaagaatacgaccaccta ggcc
TC cb32_5	cgcctaaaacataccagatcgccacccgcgctttaatctggagaggtgaagaatacgaccacctaggc
TOBRA N6G5	ggtgtttcggaaactgcggttctaccgagcact
TOBRA N8B5	ggtgtttcggaaacaaactagttctaccgagcact
NEO M4	ccggcatagcttgtcctttaatggtcctatgtcg
NEO M7	cgactgcttgtcctttaatggtccagtc
PARO	ggtagaacccacagttctaccacataccaccagagacagtcttctattggcttctactgagcaggg
PARO S1	ccacataccaccagagacagtcttctattggcttctactgagcaggg
PARO S2	ccacataccaccagagacttctattgtctactgagcaggg
PARO S3	ccacataccaccagcttctattgctgagcaggg
PARO S1 UUCG	ccacataccaccagagacagtcttcgggcttctactgagcaggg
PARO S2 UUCG	ccacataccaccagagacttcggtctactgagcaggg
PARO S3 UUCG	ccacataccaccagcttcggctgagcaggg
PARO S1 dLINK	ccaccagagacagtcttctattggcttctactgagcaggg
PARO S1 3x CAA	caacaacaaccaccagagacagtcttctattggcttctactgagcaggg
PTc1	ccacataccaccagagacagtcaaacataccagatcgccacccgcgctttaatctggagaggt gaagaatacgaccaccggcttctactgagcaggg
PTc2	ccacataccaccagagacaaacataccagatcgccacccgcgctttaatctggagaggtgaag aatacgaccaccgtctactgagcaggg
PTc3	ccacataccaccagcaaacataccagatcgccacccgcgctttaatctggagaggtgaagaat acgaccaccgctgagcaggg
PTc4	ccacataccaccagcctaaaacataccagatcgccacccgcgctttaatctggagaggtgaag aatacgaccacctaggctgagcaggg
PTc5	ccacataccaccacctaaaacataccagatcgccacccgcgctttaatctggagaggtgaaga atacgaccacctaggtgagcaggg
PTc6	ccacataccaccactaaaacataccagatcgccacccgcgctttaatctggagaggtgaagaat acgaccacctagtgagcaggg
PTb1	ccacataccaccagtgtttcggaaactgcggttctaccgagcattgagcaggg
PTb2	ccacataccaccagagacagtcttcggtgtttcggaaactgcggttctaccgagcactttggcttcta ctgagcaggg
PTb3	ccacataccaccagagacagtcttctgtttcggaaactgcggttctaccgagcattggcttctactga gcaggg
PTb4	ccacataccaccagagacagtccaacaacaaggtgtttcggaaactgcggttctaccgagcact

	caacaacaaggcttctactgagcaggg
PTb5	ccacataccaccagagaccaacaacaaggtgtttcggaaactgcggttctaccgagcactcaa<br>caacaagtctactgagcaggg
PTb6	ccacataccaccagccaacaacaaggtgtttcggaaactgcggttctaccgagcactcaacaac<br>aagctgagcaggg
PN1	ccacataccaccagagacagtgcttgtcctttaatggtccgcttctactgagcaggg
PN2	ccacataccaccagagagcttgtcctttaatggtcctctactgagcaggg
PN3	ccacataccaccaggcttgtcctttaatggtccctgagcaggg
PN4	ccacataccaccagagacagtccaacaacaagactgcttgtcctttaatggtccagtccaacaac<br>aaggcttctactgagcaggg
PN5	ccacataccaccagagaccaacaacaagactgcttgtcctttaatggtccagtccaacaacaagtct<br>actgagcaggg
PN6	ccacataccaccagccaacaacaagactgcttgtcctttaatggtccagtccaacaacaagctgagc<br>aggg
PN7	ccacataccaccagactgcttgtcctttaatggtccagtctgagcaggg
PN8	ccacataccaccaactgcttgtcctttaatggtccagttgagcaggg
PN9	ccacataccaccactgcttgtcctttaatggtccagtgagcaggg
TN1	cagtaatctgcaaacataccttagcttgtcctttaatggtcctaaggagaggtgaagaatacgaccac<br>cgcagattact
TN2	agtaatctgcaaacatacttagcttgtcctttaatggtcctaagagaggtgaagaatacgaccaccgc<br>agattact
TN3	cagtaatctgcaaacatactagcttgtcctttaatggtcctagagaggtgaagaatacgaccaccgc<br>agattact
TN4	ctcgcgagcaaacatactagcttgtcctttaatggtcctagagaggtgaagaatacgaccaccgct<br>cgcga
TN5	cggcctaaaacatactagcttgtcctttaatggtcctagagaggtgaagaatacgaccacctaggcc
TN6	cgcctaaaacatactagcttgtcctttaatggtcctagagaggtgaagaatacgaccacctaggc
TN7	cagtaatctgcaaacatacgactgcttgtcctttaatggtccagtcgagaggtgaagaatacgacca<br>ccgcagattact
TN8	cagtaatctgcaaacatacactgcttgtcctttaatggtccagtgagaggtgaagaatacgacca<br>ccgcagattact
TN9	cagtaatctgcaaacatacctgcttgtcctttaatggtccaggagaggtgaagaatacgaccacc<br>gcagattact
TN10	ctcgcgagcaaacatacctgcttgtcctttaatggtccaggagaggtgaagaatacgaccaccg<br>ctcgcga
TN11	cggcctaaaacatacctgcttgtcctttaatggtccaggagaggtgaagaatacgaccacctaggcc
TN12	cgcctaaaacatacctgcttgtcctttaatggtccaggagaggtgaagaatacgaccacctaggc
TcTb1	cagtaatctgcaaacatacggtgtttcggaaactgcggttctaccgagcactgaga<br>ggtgaagaatacgaccaccgcagattact
TcTb2	cagtaatctgcaaacataccagatcgccaaaaacaacaacaaggtgttt<br>cggaaactgcggttctaccgagcactcaacaacaatttttacccgcgctttaat<br>ctggagaggtgaagaatacgaccaccgcagattact
TcTb3	cagtaatctgcaaacataccagatcgccaaaaacaacaacaaggtgttt<br>cggaaacaaactagttctaccgagcactcaacaacaatttttacccgcgcttta<br>atctggagaggtgaagaatacgaccaccgcagattact
TcTb4	caacaacaaagtaatctgcaaacataccagatcgccaaaaacaaca<br>acaaggtgtttcggaaacaaactagttctaccgagcactcaacaacaatttttaccc<br>gcgctttaatctggagaggtgaagaatacgaccaccgcagattact
TcTb5	caacaacaaagtaatctgcaaacataccagatcgcccaaaaacaac<br>aacaaggtgtttcggaaacaaactagttctaccgagcactcaacaacaatttttgac

	ccgcgctttaatctggagaggtgaagaatacgaccaccgcagattact
TcTb6	caacaacaaagtaatctgcaaacataccagatcgccaacaacaagg tgtttcggaaacaaactagttctaccgagcactcaacaacaagcgctttaatctgg agaggtgaagaatacgaccaccgcagattact
TcTb1 F5	ctcgcgagcaaacatacggtgtttcggaaactgcggttctaccgagcact gagaggtgaagaatacgaccaccgctcgcga
TcTb1 cb32	cggcctaaaacatacggtgtttcggaaactgcggttctaccgagcactgagagg tgaagaatacgaccacctaggcc
TcTb1 cb32_5	cgcctaaaacatacggtgtttcggaaactgcggttctaccgagcactgagaggt gaagaatacgaccacctaggc
TbTc1	ggtgtttcggacttaaacataccagatcgccacccgcgctttaatctggagaggt gaagaatacgaccaccaagctaccgagcact
TbTc2	ggtgtttcggaagcaaacataccagacgccacccgcgctttaatctggagaggt gaagaatacgaccaccgctctaccgagcact
TbTc3	ggtgtttcggaactaaacataccagatcgccacccgcgctttaatctggagaggt gaagaatacgaccaccagtctaccgagcact
TbTc4	ggtgtttcggagttaaacataccagatcgccacccgcgctttaatctggagaggt gaagaatacgaccaccagcctaccgagcact
TbTc5	ggtgtttcggactttaaacataccagatcgccacccgcgctttaatctggagaggt gaagaatacgaccaccaaagctaccgagcact
TbTc6	ggtgtttcggagagcaaacataccagatcgccacccgcgctttaatctggagaggt gaagaatacgaccaccgttcctaccgagcact
TbN1	ggtgtttcggatggcttgtcctttaatggtcccactaccgagcact
TbN2	ggtgtttcggattgcttgtcctttaatggtccacctaccgagcact
TbN3	ggtgtttcggattggcttgtcctttaatggtcctaactaccgagcact
TbN4	ggtgtttcggattcgcttgtcctttaatggtccttactaccgagcact

Table S3 – Supplementary hybrid constructs dynamic ranges

Construct	Relative fluorescence*		Dynamic range at 250 µM		
	wo	both	1st ligand	2nd ligand	both
**PN7**	17.6	1.8	2.6x	7.0x	9.8x
**PN8**	17.7	2.7	2.5x	4.3x	6.6x
**PN9**	16.1	2.7	2.1x	3.8x	6.0x
**TN7**	13.5	1.4	5.0x	3.3x	9.6x
**TN8**	13.3	1.2	6.3x	2.5x	11.1x
**TN9**	12.1	0.7	10.1x	3.3x	17.3x
**TN10**	9.4	0.7	10.4x	4.7x	13.4x
**TN11**	5.0	0.8	4.2x	2.4x	6.3x
**TN12**	37.7	18.1	1.0x	2.2x	2.1x
**TcTb1**	12.0	0.8	5.7x	8.6x	15.0x
**TcTb1 F5**	9.8	0.7	7.0x	8.9x	14.0x
**TcTb1 cb32**	20.0	1.4	3.0x	10.0x	14.3x
**TcTb1 cb32_5**	19.7	1.5	2.1x	10.9x	13.1x

* Relative GFP fluorescence of hybrid riboswitches in the absence (wo) and presence of 250 µM of both of their respective ligands. A background was subtracted (pCBB06) and GFP expression was normalized to mCherry expression. The positive control under the same ligand condition as each measurement was set to 100%. Measurements were performed in triplicates and repeated twice.
